# Polymer Matrices for Reversible Thermogelling Hydrogels: Principles, Fabrication, and Drug Delivery Prospects

**DOI:** 10.3390/polym18060681

**Published:** 2026-03-11

**Authors:** Victor S. Pyzhov, Elena O. Bakhrushina, Vladimir I. Gegechkori, Valery V. Smirnov, Grigoriy Y. Evzikov, Anna K. Kartashova, Irina M. Zubareva, Ivan I. Krasnyuk, Ivan I. Krasnyuk

**Affiliations:** Ministry of Health of the Russian Federation, Sechenov First Moscow State Medical University, 8-2 Trubetskaya Str., Moscow 119991, Russia

**Keywords:** thermosensitive hydrogel, LCST, in situ gel, drug delivery, poloxamer, chitosan, xyloglucan, PLGA-PEG, PNVCL, polyoxazoline, elastin-like polypeptides

## Abstract

This review presents a comprehensive analysis of modern thermosensitive polymer systems for in situ systems (ISSs) which are used for targeted drug delivery in situ. The main classes of polymers used to create “smart” hydrogels that undergo a “sol–gel” phase transition in response to a temperature stimulus in the physiological range are considered. Key representatives of thermosensitive matrices are described in detail: synthetic block copolymers (poloxamers, block copolymers of polylactic-co-polyglycolic acid with polyethyleneglycol, etc.) and natural, modified natural, and semi-synthetic polymers (chitosan, including in combination with β-glycerophosphate, xyloglucan, etc.). This paper systematizes the advantages and disadvantages of various thermosensitive systems and highlights the key risks in their pharmaceutical development, including the influence of the nature and concentration of the active pharmaceutical ingredients and excipients on the rheological properties and phase transition temperature. Particular attention is paid to the difference between thermoreversible and irreversible gel-forming systems. Modern in vitro, ex vivo, and in vivo methods for evaluating critical quality parameters of thermosensitive systems, such as gelation temperature and time, gel strength, mucoadhesive properties, and release kinetics, are discussed. The need to develop standardized and biologically relevant methods to improve the reproducibility and success of preclinical studies is emphasized. The review is intended to help researchers to make informed choices about polymer matrices and optimize compositions for successful pharmaceutical development.

## 1. Introduction

In modern pharmaceutical technology, drug delivery systems based on stimulus-sensitive polymers are becoming increasingly popular. Such systems are called in situ delivery systems or just in situ systems (ISSs). In situ is Latin for “in place,” and in drug technology, this term is used to refer to stimulus-dependent systems that can undergo a phase transition at a specific location in the body due to physiological or pathological factors and the targeted and modified (prolonged or accelerated) release of the active pharmaceutical ingredient (API). In a more specific sense, ISSs are liquid reversible systems that undergo a solution–gel (sol–gel) transition locally in the target organ under the influence of thermal, chemical, or physicochemical factors related to the physiological conditions of the environment of application [1,2].

According to the PubMed database, the first mention of the term “in situ” in the scientific literature in the context of local gel formation dates back to 1984 and describes the formation of gelatin gel from its aqueous solution in the stomachs of Wistar rats following a hydrated gelatin diet [3].

With the development of the chemical and petroleum industries, the range of polymer excipients for pharmaceutical development gradually increased, which significantly expanded the possibilities for creating modified-release drug delivery systems. The first scientific developments of in situ systems date back to the late 20th and early 21st centuries. In 1999, a group of authors led by B. Cheng published a paper on the development of an innovative biodegradable polymer of polylactic-co-glycolic acid, the aqueous solutions of which are capable of undergoing a reversible thermosensitive transition from solution to gel at human body temperature [4].

Based on their origin, polymers used in pharmaceutical technology in situ drug delivery systems are divided into the following types:

(1) Natural (chitosan, gellan gum, pectin, shellac, etc.) [5];
(2) Synthetic (block copolymers of ethylene oxide and propylene oxide (poloxamers), polylactic-co-glycolic acid and its copolymers with polyethylene glycol, polycaprolactone, etc.) [6];
(3) Semi-synthetic polymers (modified forms of chitosan, xyloglucan, etc.) [6,7,8].

Depending on the type of stimulus, the matrices of ISSs can be divided into the following types: thermoreversible/thermosensitive; pH-dependent; ion-sensitive; moisture-activated; phase-sensitive; photosensitive; and, according to new data, sensitive to redox stimuli and enzymes [6,7,8,9].

ISSs are already actively used in various fields of medicine, such as ophthalmology, dentistry, oncology, and gynecology. Stimulus-sensitive smart systems composed of hydrogels stand out among other drug delivery systems and other dosage forms with prolonged drug release in many respects:

(1) The possibility of their injection administration, without surgical intervention, as in the case of implants.
(2) Simple manufacturing technology, requiring only final sterilization, compared to systems containing liposomes, nanoparticles, or microspheres with the API [10,11,12].
(3) Targeted drug delivery: These drug delivery systems can be administered subcutaneously or intravenously directly to the desired site of application, where, under the influence of a specific factor, a stable gel network forms, from which the active substance is released [13,14].
(4) The ability to regulate the rate of release of the active substance from the matrix by including polymeric excipients with specified properties in the system [15].
(5) High bioavailability of drugs due to bypassing first-pass metabolism (hepatic metabolism) [14].

## 2. Materials and Research Methods

This review was conducted to provide a comprehensive and critical analysis of thermosensitive polymer matrices used for reversible in situ-forming hydrogels in drug delivery applications. A literature search was performed using the following electronic databases: Scopus (www.scopus.com), Web of Science (www.webofscience.com), PubMed (www.pubmed.ncbi.nlm.nih.gov), Google Scholar (www.scholar.google.com), and the Russian scientific electronic library eLibrary (www.elibrary.ru). All databases were accessed between January and November 2025.

The keywords used in the search strategy included: “thermosensitive hydrogel,” “LCST,” “lower critical solution temperature,” “in situ gel,” “injectable gel,” “drug delivery,” “controlled release,” “poloxamer,” “Pluronic,” “chitosan,” “xyloglucan,” “PLGA-PEG,” “PNVCL,” “poly(N-vinylcaprolactam),” “poly(2-oxazoline),” “polyoxazoline,” and “elastin-like polypeptide.” Boolean operators (AND/OR) were applied to combine terms and maximize retrieval of relevant studies (e.g., “thermosensitive hydrogel” AND “LCST,” (“poloxamer” OR “Pluronic”) AND “drug delivery”).

The search covered the period from January 1990 to February 2025, with a particular focus on publications from the last 10 years to ensure inclusion of recent advances. No restrictions were applied regarding study design during the initial search phase to ensure comprehensive coverage.

The selection was limited to full-text studies published in English or Russian. The following inclusion criteria were applied: original research articles and review articles focusing on thermosensitive polymer matrices for medical applications, with clear specification of gelation mechanisms, LCST parameters, and biocompatibility or cytotoxicity data. All relevant study types investigating thermosensitive hydrogels for drug delivery applications were considered for inclusion.

The following exclusion criteria were applied: studies lacking experimental data; works dedicated solely to polymer synthesis without investigation of pharmaceutical or biomedical properties; publications in languages other than English or Russian; conference abstracts without accompanying full-text articles; and studies not reporting temperature-responsive behaviour or LCST-related parameters.

All articles retrieved from the databases were recorded in an internal dataset and subjected to a two-stage selection process. In the first stage, titles and abstracts were analyzed independently by two reviewers to determine relevance based on the predefined inclusion criteria. Conflicts regarding study eligibility were resolved through discussion or by consultation with a third reviewer. In the second stage, the full text of articles of potentially eligible studies was retrieved and further assessed independently by two reviewers against the same inclusion and exclusion criteria. Reasons for exclusion at this stage were documented (e.g., “no LCST data reported,” “synthesis-only paper without biological evaluation,” “language not English/Russian,” “duplicate publication”).

From each eligible study, the following information was extracted using a standardized data extraction form: polymer type and class, gelation mechanism, LCST or phase transition temperature range, experimental methods used (e.g., rheology, differential scanning calorimetry), gelation parameters (time, strength), drug model (if applicable), release profile data, biocompatibility or cytotoxicity results, and any reported limitations or challenges.

**Figure 1:**
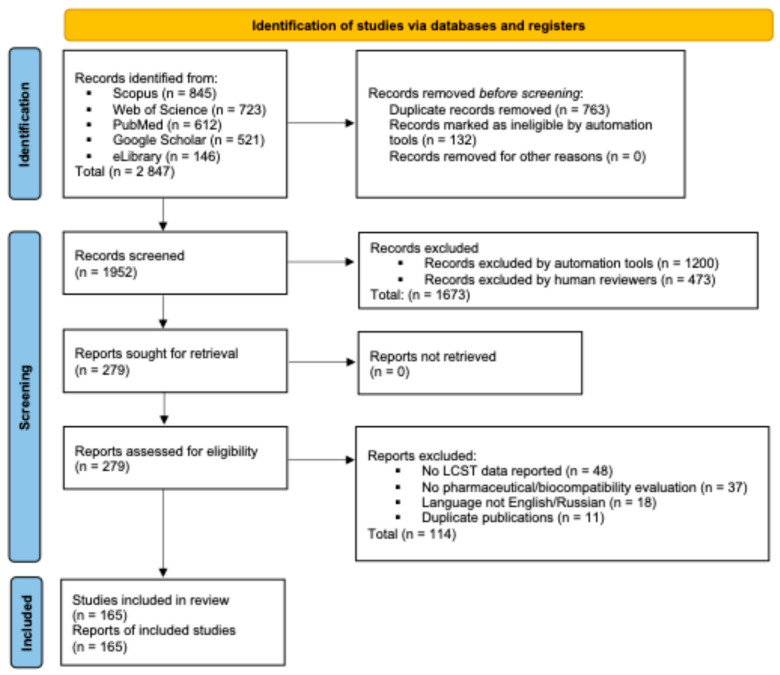
PRISMA flow diagram.

A total of 2847 studies were screened from January 1990 to February 2025. After removal of duplicates (*n* = 895), 1952 records were screened by title and abstract. Of these, 1673 records were excluded as they did not meet the inclusion criteria (e.g., unrelated to thermosensitive hydrogels, no drug delivery application, synthetic chemistry focus only). The full texts of the remaining 279 articles were assessed for eligibility. Following full-text review, 114 articles were excluded for reasons including absence of LCST data (*n* = 48), lack of pharmaceutical or biocompatibility evaluation (*n* = 37), language restrictions (*n* = 18), and duplicate or overlapping publications (*n* = 11). A total of 165 studies met all inclusion criteria and were included in this review. A flow chart summarizing the search and selection process has been added as Figure 1 to provide a transparent overview of study inclusion.

## 3. Main Part

### 3.1. Thermosensitive Matrices for In Situ Systems—A Retrospective Study

One of the most studied and promising areas of development for ISSs in terms of stimulus versatility and control is the development of thermoreversible smart systems. These systems include thermosensitive or thermoreversible polymers or combinations thereof, most often dissolved in water as the most biocompatible and non-toxic solvent. They are usually fluid, low-viscosity solutions at temperatures up to 25 °C, which, when introduced into the body, are capable of undergoing a solution–gel transition under the influence of body temperature, forming a drug depot in the target organ or directly at the site of application [16].

Thermosensitive ISSs have all the advantages of stimulus-sensitive systems and a number of significant features that distinguish them from other smart systems:

(1) Due to the relatively high rate of temperature-sensitive transition and fluidity, these hydrogels are able to take the shape of the area into which they are introduced, which ensures a uniform rate of release of the active substance in the target organ [13,14].
(2) The release of the active pharmaceutical substance from the thermogel matrix is practically unaffected by the pH of the environment, its ionic composition, or the presence of certain biologically active substances (enzymes, inflammatory mediators, etc.), by which thermoreversible systems ensure the stable and sustained release of active substances at the site of application. This property is often critical in the treatment of chronic diseases [17].
(3) The water content in the thermogel matrix contributes to its biocompatibility and allows it to be used as a system for drug delivery and moisturizing sensitive areas of the body [18].
(4) Most thermogels are easily manufactured using aqueous solvents and are physically crosslinked due to the effect of ambient temperature. This method avoids the use of organic solvents or additional crosslinking agents that may have their own pharmacological or toxic effects. In particular, this makes it possible to use thermosensitive matrices as transporters of proteins, peptides, and other immunobiological substances, reducing the risk of their denaturation or degradation [19,20].

The first mention of thermosensitive polymers in the scientific community appeared in 1965 in the Journal of the American Oil Chemists Society, where a group of authors led by Irwing R. Schmolka published a study on the properties of block copolymers of polyethylene oxide with polypropylene oxide, which later received the trade name Pluronic [21].

Subsequently, as polymers with similar properties were developed and studied, a reference thermoreversible polymer, poly(N-isopropylacrylamide), was synthesized, and its popularity to this day is due to its ability to undergo a reversible thermosensitive transition within the physiological temperature range [22].

A landmark event in the development of thermoreversible matrices for medical applications was the FDA’s approval of Pluronic F-68 in 1998. Subsequently, research in this area shifted towards the development of thermosensitive matrices that are not only biocompatible and non-toxic to humans but also capable of enzymatic biodegradation in the human body. A 1994 study presented block copolymers of polylactic-co-glycolic acid with polyethylene glycol capable of biodegradation in the human body [23]. By the beginning of the 21st century, many thermosensitive polymers had been developed and studied, and the mechanism of their phase transition in accordance with the Flory–Huggins law had been substantiated. Methods for the synthesis or modification of polymer matrices, and modulation of natural polymers were further developed to give these materials thermosensitive properties in physiological temperature ranges.

### 3.2. Mechanism of Thermosensitive Transition

To create effective smart systems based on thermosensitive polymers, it is necessary to understand the molecular mechanism underlying their phase transition [24]. The behavior of thermoreversible polymers in solutions can be explained by several thermodynamic models, the first of which is Flory’s theory, which states that the statistical properties of chains in a polymer melt coincide with the properties of ideal chains. According to this theory, a polymer in a solution or melt is considered as a chain of structurally linked fragments, each of which influences neighboring elements. At the same time, the direction and force of repulsion of the links as they approach each other are always compensated by an equal distance from other fragments of the polymer chain, thus maintaining thermodynamic equilibrium within the polymer chain [25].

According to the Flory–Huggins theory, all polymers can be divided into two groups based on their interaction with solvents:

(1) Polymers with an upper critical solution temperature (UCST). Delamination in solutions of such polymers occurs in temperature ranges before and after the UCST, and, when this temperature is reached, the most effective polymer–solvent interaction is observed (e.g., polystyrene in cyclohexane and acetylcellulose in chloroform). Polymer matrices, the phase transition of which is determined by the upper critical point of dissolution, are practically unused in pharmaceutical hydrogel technology since a temperature above 37–39 °C is generally required to achieve fluidity in such systems. Nonpolar solvents with either their own pharmacological action or toxicity are instead used, which significantly complicates the design of hydrogel systems [26].
(2) Polymers with a lower critical solution temperature (LCST). In cases with an LCST in aqueous polymer solutions, the renormalization of polymer–polymer interactions occurs due to the interaction of chain fragments with the solvent. An increase in temperature to the LCST causes an increase in hydrophobic interactions within the polymer chain and between its molecules, but the thermodynamic stability of the system decreases due to a reduction in the entropy of water caused by a disruption in its hydrogen bond network (e.g., aqueous solutions of polyethylene oxide (PEO), poly-N-isopropylacrylamide (PNIPAA), and others) [16].

### 3.3. Polymer Systems with LCST

Currently, the study and development of compositions based on new polymers capable of undergoing a solution–gel phase transition in aqueous solutions is a priority area in the pharmaceutical development of hydrogel drug delivery systems.

**Figure 2:**
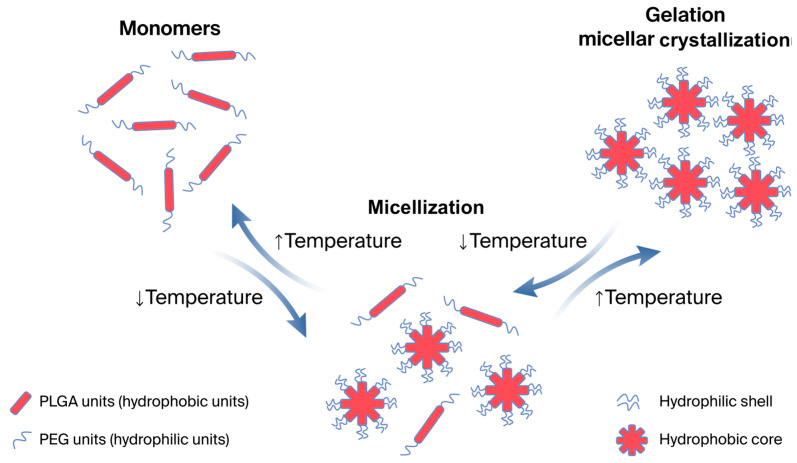
Mechanism of thermoreversible gelation (PLGA–PEG as example).

We consider the molecular and conformational changes in aqueous solutions of PNIPAAM, a clear example of a polymer with an LCST. Poly(N-isopropylacrylamide) (PNIPAAM) has been extensively researched and is one of the most well-studied thermosensitive polymers. The LCST of PNIPAAM in hydrogel ranges from 32 °C to 34 °C [27,28,29,30]. In solutions, this polymer demonstrates a gradual transition from a helix to a globule as the temperature rises to the LCST, thus increasing intramolecular stability due to hydrophobic interactions between the links of the polymer chain [31,32,33]. Hydrogen bonds between the hydrophilic segments of the polymer chains and water molecules prevail below the LCST in the aqueous solution system of PNIPAM, which leads to an increase in its solubility and swelling rate. As the temperature rises and approaches the LCST, the number of hydrophobic interactions between hydrophobic segments increases, and hydrogen bonds gradually become thermodynamically unstable and break down [27,34]. Figure 2 is an illustration of the mechanism of the thermoreversible gelation of polymers with the LCST.

Another polymer with an LCST is a triblock copolymer of polyethylene oxide (PEO) and polypropylene oxide (PPO), which has the ability to undergo a thermoreversible transition. Its aqueous solutions are liquid at low temperatures and reversibly transform into a gel when the temperature rises. The speed and temperature of the transition depend on the ratio of hydrophilic (PEO) and hydrophobic (PPO) elements in the polymer chain and on the concentration of the macromolecular compound in the solution. When the temperature rises to the critical gelation temperature (CGT) and the polymer concentration rises to the critical micelle concentration (CMC), the hydrophobic elements tend to dehydrate and self-assemble, first into aggregates then into micelles (micellization). The mechanism of micellization of such polymers obeys the Anionsson–Wall model, which describes the dynamics of self-assembling systems. According to this model, when the CMC is reached, hydrophobic elements form the core of the micelle, while hydrophilic elements line up at the periphery in the form of a micelle crown. The geometry of micelles can be spherical or worm-like, depending on the composition of the poloxamer. The transition to a worm-like (linear) shape is influenced by gradual micellar growth, which occurs due to the fragmentation of micelles and their aggregation [35].

In addition to the above-mentioned polymers, the literature describes the use of block copolymers of polyethylene glycol with caprolactone [36], PLGA [37], polylactide [38], polyacrylates [39], as well as natural macromolecular substances of polysaccharide or protein origin [40,41,42].

In general terms, the mechanism of the stimulus-sensitive solution–gel transition of solutions of high-molecular compounds consists of several main stages:

(1) Swelling of the polymer in an aqueous medium (hydration of hydrophobic and hydrophilic groups of a high-molecular compound with the formation of hydrogen bonds) and its further dissolution.
(2) When exposed to a stimulus, the hydrophobic links dehydrate and tend to undergo intermolecular hydrophobic interaction and aggregation due to the breaking of hydrogen bonds with water. This process can be considered micelle formation.
(3) When the solution reaches the critical gelation temperature, the formed micelles bind into structures of various geometric shapes and ultimately form a gel network due to the interaction between the hydrophilic elements of the micelle crown (due to hydrogen bonds and van der Waals interactions) [43].

### 3.4. Thermosensitive Hydrogels with LCST and Crosslinking Agents

In general, hydrogels are three-dimensional polymer networks capable of swelling in an aqueous environment to several times their original weight. This makes them widely used in a variety of fields, from the food and cosmetics industries to modern areas such as cell cultures, drug delivery, and tissue engineering [44,45,46,47]. Thermosensitive hydrogels are in a liquid state but can undergo a phase transition to a viscoelastic or even solid form under the influence of temperature manipulation.

There are two basic ways to obtain stimulus-sensitive compositions from thermosensitive polymers:

(1) Physical crosslinking: In this phenomenon, the formation of a hydrogel from a solution occurs without the introduction of additional crosslinking agents (crosslinkers) into the composition, solely under the influence of physical stimuli such as temperature, the pH of the medium, UV radiation, or a change in solvent.
(2) Chemical crosslinking: In some polymer compositions, the addition of crosslinkers is required to achieve a stimulus-sensitive solution–gel transition. These crosslinkers interact with the polymer, modifying its side groups, linking them into a single polymer network through the formation of covalent bonds, or acting as activators of ion-sensitive or thermosensitive transitions [48,49,50].

Low-molecular-weight compounds such as glutaraldehyde, diglycidyl ether, diisocyanates, or diacrylates are often used as crosslinkers. They can be used not only to impart stimulus-sensitive properties to the hydrogel but also to regulate the effect of the stimulus under study. For example, in thermosetting compositions of individual PNIPAAM, its LCST ranges from 31 °C to 33 °C, which is slightly below the physiological temperature range. However, when N,N′-methylene-bis-acrylamide or N,N′-cystamine-bis-acrylamide crosslinking agents are added to its aqueous solution, the temperature of its phase transition can be shifted to the range of 36–38 °C [51]. It should be noted that the addition of crosslinking agents to a pharmaceutical composition always carries the risk of additional pharmacological or toxic effects due to the properties of the crosslinker [52,53,54].

Another example of the use of crosslinking agents is the combination of chitosan with beta-glycerophosphate (βGP). βGP in compositions with chitosan acts as a pH stabilizer and crosslinker, giving chitosan thermosensitive properties. The hydrogel based on this composition is accordingly capable of undergoing a reversible solution–gel transition within 5 min at a temperature of 37 °C, provided that the individual chitosan is not prone to thermoreactivity [55].

In addition to modifying stimulus sensitivity, crosslinking agents are used in thermosensitive hydrogel compositions to modulate the release of the API from the delivery system matrix or to impart additional pharmacological properties to it. For example, there is a known thermoreversible system based on a three-block copolymer, PEG–poly(ε-caprolactone)–PEG (PEG–PCL–PEG), synthesized via ring-opening polymerization, which demonstrates a solution–gel transition at 37 °C [56]. Injectable hydrogels based on this system are actively used in tissue engineering, and, in the work of a team of authors led by X. Zhao, an aniline tetramer was grafted onto PEG–PCL–PEG to improve its tissue-regenerative properties using electrostimulation [57].

### 3.5. Thermosensitive Hydrogels Based on Polysaccharides

#### 3.5.1. Chitosan/Beta-Glycerophosphate

Chitosan is a natural polysaccharide with a straight chain of (1 → 4)-linked 2-amino-2-deoxy-D-glucopyranose with some residual D-glucosamine units [58].

In general, chitosan is practically insoluble at physiological pH values, but, in diluted acidic solutions with a pH of less than 6, the primary amino groups of chitosan convert to a protonated form, resulting in the formation of a water-soluble polycation. The polycationic nature of chitosan also determines its interaction with the negatively charged cell membranes of microorganisms and explains its antimicrobial effects and mucoadhesive properties [59]. Acetic and formic acids are the two most widely used acids for dissolving chitosan. Some diluted inorganic acids (e.g., nitric, hydrochloric, chloric, and phosphoric) can also be used to prepare a chitosan solution but only after prolonged stirring and heating. However, in some cases, a white gel-like precipitate forms after the dissolution of the polymer in a nitric acid solution. Mixtures such as dimethylformamide with nitrogen tetroxide in a 3:1 ratio are also good solvents for chitosan [59].

Thus, aqueous chitosan solutions are not capable of temperature-sensitive sol–gel transition while chitosan precipitates from solution upon heating without the addition of crosslinking agents to the composition or without modification of the molecules of the polymer. The presence of a large number of amino groups and hydroxyl groups allows for an improvement of the physicochemical properties of chitosan.

**Figure 3:**
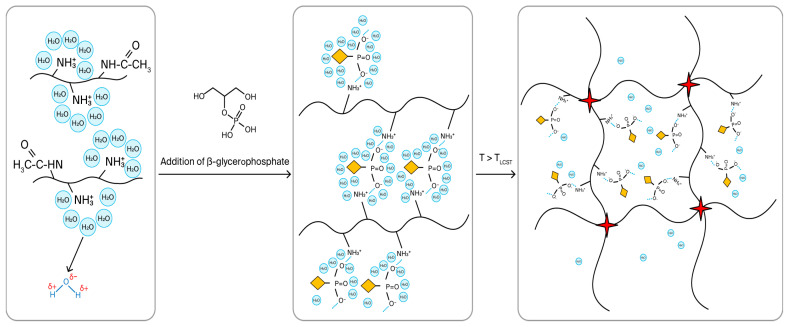
Mechanism of thermoreversible gelation of chitosan/β-glycerophosphate (or other polyol-phosphate) compositions.

Researchers are most interested in developing a thermosensitive in situ system based on chitosan and β-glycerophosphate (βGP) as a crosslinking agent. Figure 3 demonstrates the mechanism of thermoreversible gelation of chitosan/β-glycerophosphate (or other polyol-phosphate) compositions.

In 2000, a team of authors led by Chenite developed a thermosensitive hydrogel system based on chitosan by adding β-glycerophosphate. β-glycerophosphate raises the pH of acidified chitosan to 7–7.4, acting as a weak base salt, which allows this composition to be injected without hindrance. At the same time, βGP gives chitosan thermosensitive properties, allowing the composition to reversibly form a gel when it reaches 37 °C and return to a liquid, non-viscous state at 10 °C. The solution obtained by the authors was a transparent liquid at room temperature but immediately gelled at physiological body temperature [60,61].

The mechanism of thermoreversibility of the chitosan–β-glycerophosphate system can be explained as follows:

(1) βGP reduces the polarity of chitosan amino groups, which leads to an increase in the hydrophobicity of the polymer matrix.
(2) Due to the increase in the hydrophobicity of the polymer chain, the degree of chitosan solvation decreases, and dehydration of the chitosan chain occurs.
(3) Electrostatic attraction occurs between the chitosan and β-glycerophosphate chains due to the transfer of protons from the polymer amino groups to β-glycerophosphate, which leads to the formation of hydrogen bonds and the establishment of thermodynamic equilibrium [60,62].

To establish thermodynamic equilibrium in the system, a balance must be established between the following forces: (1) the force of electrostatic repulsion within the chitosan molecule, (2) the force of attraction between the positively charged amino groups in chitosan and the negatively charged phosphate group in β-glycerophosphate, (3) the force of hydrogen bonds and hydrophobic interactions arising between chitosan fragments, and (4) the ability of βGP polyol fragments to reduce the surface tension at the water–chitosan phase boundary [60].

In addition to the above, Cho and colleagues attribute the occurrence of a thermoreversible transition in the chitosan–β-glycerophosphate system to a decrease in the number of hydrogen bonds between individual chitosan chains, which leads to a more orderly formation of hydrophobic micellar nuclei as the temperature in the solution increases. This change introduced by βGP into the system allows the formation of stable chitosan micelles in a solution with a branched hydrophilic crown, which is capable of forming a large number of intermolecular hydrogen bonds, forming a stable gel network at physiological temperature [63].

It is worth noting that βGF is used to neutralize the excessively high pH value of the phase transition. Thus, thermosensitive chitosan-based injectable hydrogels, which include disodium βGP used for pH neutralization, have a gelation temperature of about 37 °C. This is explained by the formation of hydrogen bonds between chitosan chains due to a decrease in repulsive forces (due to the basicity of the salt) and further hydrophobic interactions during temperature increase [64]. Although the use of beta-glycerophosphate-neutralized hydrogels is beneficial, it has been reported that high concentrations of βGP are required to achieve physiological pH levels, which reduces the cytocompatibility of the composition [63].

The main challenges in developing an injectable smart system based on chitosan with β-glycerophosphate (βGP) are as follows:

(1) Reproducibility and raw material standardization. The properties of chitosan (molecular weight, degree of deacetylation, distribution of acetyl groups, and impurity content) are critically dependent on the raw material source (crustacean shells or fungal mycelium) and the production method. This leads to high variability in key parameters of the final system: gelation temperature, gelation rate, gel strength, and drug release kinetics [65]. The absence of pharmacopeia monographs for chitosan with stringent specifications for medical use exacerbates the problem.
(2) Sterilization challenges. Like most thermosensitive systems, the chitosan/βGP composition cannot be sterilized by autoclaving. Sterilization via filtration (0.22 µm) is only possible for pre-purified, low-viscosity solutions. Sterilization with gamma irradiation can depolymerize and alter the properties of chitosan. The most reliable, yet also the costliest, method remains aseptic manufacturing [66].
(3) Long-term physical and chemical stability. During storage, the following may occur: (1) gel syneresis (separation of the liquid phase); (2) gradual pH change; (3) slow depolymerization of chitosan; and (4) interaction of the active pharmaceutical ingredient with the amino groups of chitosan. These factors necessitate thorough stability studies in accordance with national regulatory requirements and ICH guidelines.
(4) Issues of complete biodegradation and safety. Although chitosan is considered biodegradable due to the effect of lysozyme and chitinases, the rate and completeness of its in vivo degradation, as well as the impact of degradation products (oligomers, N-acetylglucosamine) on long-term safety and local immune response, are insufficiently studied [67].

Nevertheless, the chitosan/βGP combination remains a scientifically and practically relevant platform for the development of injectable smart systems.

#### 3.5.2. PEGylated Chitosan

Another method of forming thermosensitivity in chitosan is to modify its side chains with amino groups using polyethylene glycols (PEGs). In a study by Cao and colleagues [68], chitosan was modified via the attachment of methoxy-poly(ethylene glycol) (mPEG) through the formation of imine bonds with their subsequent reduction, resulting in mPEG-g-chitosan. This gave the polymer more pronounced hydrophilic properties. During the experiments, the authors noted the easy flowability of the solution at a temperature of 4 °C. However, at a temperature of 37 °C, a 1.5% solution of mPEG-g-chitosan turned into a viscous gel, which was difficult to draw up with a syringe, and significant air bubbles formed during injection.

When considering the mechanism of thermally dependent gel formation in this system, it is important to note that mPEG-g-chitosan chains are randomly distributed in space at low temperatures (4 °C) and are dominated by hydrophilic interactions, while, at higher temperatures (37 °C), the added thermal energy contributes to an increase in entropy in the system, which leads to a conformational rearrangement of the polymer chain. This leads to a solution–gel transition due to the dehydration of nonpolar polymer links and an increase in hydrophobic interactions. At the same time, mPEG-g-chitosan chains form aggregates that gradually build up into a gel network. However, an increase in the concentration of mPEG-g-chitosan in the solution disrupts the formation of the gel network. It is also important to note that repulsion occurs between the similarly charged protonated amino groups of mPEG-g-chitosan when the pH of the system falls below the pKa of chitosan. Thus, the hydrophobic interactions between polymer chains necessary for packing and gel network formation are reduced due to electrostatic repulsive forces between partially protonated amino groups of chitosan, and gel formation is hindered due to a decrease in intermolecular interactions of polymer chains [64].

Thermosensitive systems based on chitosan derivatives have good prospects for use in further clinical practice. There is much more information available about these systems and the tests conducted with them than for pH-sensitive in situ systems. The ability of some systems to undergo reversible gelation without reducing stability is beneficial for both the manufacturing process and handling and is particularly worth emphasizing. Several studies report satisfactory viscosity values for the compositions obtained and, more importantly, phase transition temperatures within physiological limits, which will certainly accelerate further research into chitosan-based thermosensitive systems.

The challenges in developing ISSs based on PEGylated chitosan are as follows:

(1) Heterogeneity of the final product. PEGylation is a statistical process that can occur at different functional groups of chitosan (primary C2 amines and primary and secondary C6 and C3 hydroxyls). This results in a mixture of isomers with varying numbers and positions of attached PEG chains, affecting the reproducibility of the LCST, gelation rates, and biodistribution [69]. The development of methods for selective PEGylation (e.g., exclusively at amino groups) is an active area of research.
(2) Sterilization and stability issues. The covalent bond between PEG and chitosan (often amide or carbamate) can be susceptible to hydrolysis at extreme pH values or during sterilization. Gamma irradiation sterilization can cause degradation of both PEG and chitosan. Careful selection of sterilization conditions (possibly aseptic manufacturing) and long-term stability studies of the final dosage form are required.
(3) Impact on mucoadhesive properties. While PEG improves solubility and biocompatibility, it can shield the positive charges of chitosan, thereby reducing its inherent mucoadhesiveness. Partial PEGylation or the introduction of additional mucoadhesive agents is sometimes employed to compensate for this effect [70].
(4) Potential immunogenicity of PEG. Recent years have seen emerging data on the formation of anti-PEG antibodies in some patients, which can lead to accelerated clearance of PEG-containing drugs and reduced efficacy upon repeated administration (the “ABC” effect—accelerated blood clearance). This risk must be considered when designing systems for chronic diseases [71].

The most promising direction is the development of “smart” dual systems, where PEGylation addresses solubility issues and imparts thermosensitivity, while non-reacted amino groups of chitosan are conjugated with targeting ligands (e.g., folic acid for targeting tumor cells). The use of biodegradable PEG analogs (e.g., based on polyglycerol or polyoxalate) may reduce the risk of long-term PEG accumulation in the body.

Thus, PEGylated chitosan represents high technology but also a high-risk platform. Its successful commercialization will depend on the developers’ ability to ensure the highest level of quality control at all production stages and to present compelling preclinical and clinical data demonstrating its superiority over existing alternatives.

### 3.6. Xyloglucan

Another noteworthy polymer for creating thermosensitive matrices is xyloglucan. Xyloglucans (XGs) represent the main class of structural polysaccharides found in the walls of primary cells of higher plants. Xyloglucan is a neutral, biocompatible polysaccharide found in the cell walls of dicotyledonous and some monocotyledonous plants. The main chain of the molecule consists of 300–3000 D-glucopyranose residues connected by β-1,4-glycosidic bonds with 1,6-α-xylosyl residues, some of which may be linked by β-3,2 bonds to D-galactopyranose residues or α-3,2 bonds to N-arabinofuranose residues. D-galactopyranose fragments may be linked to N-fucopyranose residues. In most cases, the xyloglucan molecule consists of repeating blocks with substituted and unsubstituted glucose residues. The acetylation of D-glucopyranose, D-galactopyranose, or N-arabinofuranose residues is also possible. The nature of the side branches and the order of alternation of xyloglucan elements depend on the specific plant species from which the polymer is obtained and may vary during the formation of the plant cell wall, leading to a variety of structural variants of this polysaccharide [72]. For example, xyloglucan extracted from tamarind seeds contains approximately 45% glucose, 38% xylose, 17% galactose, and a small amount of arabinose, and the polysaccharide of the Hymenaea courbaril plant contains about 40% glucose, 34% xylose, and 20% galactose [73].

Although xyloglucan is an effective gelling agent at low concentrations, in its pure form, it does not exhibit thermosensitive properties due to its developed system of side groups, which hinder conformational changes in the polysaccharide solution. To impart thermoreactive properties to xyloglucan, it is treated with an enzyme specific to glycosidic bonds, beta-galactosidase, usually obtained from Aspergillus oryzae mold fungi. This enzyme reaction degrades the glycosidic bonds between xylose and galactose residues in the side chains. Depending on the duration of the degalactosylation reaction, up to 100% of the galactose residues can be removed from the polymer, increasing its hydrophilic properties and solubility. Xyloglucan becomes temperature-sensitive when partially degalactosylated (Deg-XG): it can form physical, thermoreversible gels when the temperature in aqueous solutions changes [73,74].

Degalactosylated xyloglucans demonstrate some advantages over other thermoreversible gelation systems currently available: gelation does not require the presence of divalent cations and does not depend on the nature of the drug charge, and the gel forms within a few minutes, depending on the concentration of the polymer in the solution and the temperature [75]. The degree of degalactosylation directly affects the phase transition temperature of xyloglucan solutions, which indicates the possibility of using aqueous solutions of this polysaccharide in physiological temperature ranges [75].

For example, using xyloglucan from tamarind seeds made it possible to increase the bioavailability of rufinamide. This antiepileptic drug was characterized by low oral bioavailability, which is why high and frequent doses were necessary. The results of the study showed that the concentration of xyloglucan had a direct and significant effect on the properties of the gel and the release and kinetics of the drug. The absence of nasal toxicity is also associated with the presence of xyloglucan in the system, which has anti-inflammatory properties [76]. In the comparison of rufinamide in the in situ system with aqueous suspensions, the latter showed worse characteristics. The gel demonstrated longer exposure to the nasal mucosa and prolonged the effect of the active pharmaceutical ingredients (APIs). It was demonstrated that 90% of the total amount of nasally administered rufinamide reached the brains of rats, suggesting that the best delivery conditions for rufinamide are an intranasal, thermosensitive, in situ system.

The use of xyloglucan as a thermosensitive component in in situ systems has also been found in dental practice. Lidocaine hydrochloride was used in a xyloglucan-based delivery system to treat periodontitis. Periodontal disease is a chronic bacterial infection that affects the gums and supporting structures of the teeth. Local anesthetic gels are easy to apply, but they have disadvantages such as potential spread to other areas, reduction in the latent period, numbness in unintended areas (lips, tongue, and cheeks), and an increased likelihood of swallowing the gel. As shown by a study on the oral mucosa of sheep, gel formation occurred at 37 °C, a temperature within the physiological range of the human body. Initially, an accelerated release of the API was observed when the viscosity of the gel changed, but it was found that this led to a faster anesthetic effect. Due to the mucoadhesive properties of xyloglucan, the gel remained on the mucous membrane throughout the entire dental procedure. As a result, the release percentage of lidocaine hydrochloride was 98.05%. The use of a thermosensitive in situ system made it possible to abandon injection therapy and reduce the cost of treating the disease [77].

The main challenges in developing thermosensitive systems based on deglycosylated xyloglucan are as follows:

(1) High variability of the raw material: The properties of native xyloglucan (molecular weight distribution, monosaccharide ratio, and degree of branching) are critically dependent on the plant species, cultivation conditions, and extraction methods. This poses significant challenges for the reproducibility of the deglycosylation process and, consequently, for the stability of the gelation temperature and rheological properties of the final product [74]. The development of stringent specifications for the initial polysaccharide is required.
(2) Complexity of controlling the enzymatic process: The deglycosylation process must be carefully controlled (time, temperature, and enzyme activity) to achieve the desired degree of conversion. Stopping the reaction at the precise point and completely inactivating the enzyme require additional purification steps, which increase production cost and complexity [78].
(3) Limited mechanical strength of the gels: Gels based on Deg-XG, especially at low polymer concentrations (1–3%), may exhibit insufficient mechanical strength and resistance to erosion in dynamic biological environments (e.g., in the nasal cavity during respiration). This often necessitates the creation of composite formulations with other polymers (poloxamers or alginate), which complicates the formulation [73].
(4) Sterilization challenges: Like other thermo- and biopolymer systems, Deg-XG is sensitive to high temperatures and radiation. Gamma irradiation sterilization can cause depolymerization. Aseptic manufacturing and sterilization through filtration (for low-viscosity solutions) are the primary, yet costly, methods.

Systems based on Deg-XG represent an environmentally friendly and biocompatible alternative to synthetic polymers, particularly for applications where natural origin and mild action are important. However, their path from laboratory development to commercialization requires substantial investment in standardization and a complete cycle of preclinical and clinical trials.

### 3.7. Other Polysaccharides

Recently, a wide range of synthetically modified polysaccharides have been developed that exhibit solution–gel transition properties upon reaching the LCST. These include the following:

(1) Carboxylated agarose (~10–50 °C) [79]: This polymer is a modified polysaccharide obtained through the carboxylation of agarose chains, typically using monochloroacetic acid in an alkaline environment. While agarose itself is a polymer with a UCST (upper critical solution temperature), the introduction of carboxyl groups imparts the properties of a polyanionic high-molecular-weight compound, conferring both pH- and thermosensitivity. For instance, a known composition based on carboxymethyl agarose and poly-N-isopropylacrylamide, containing doxorubicin, forms a stable liquid hydrogel at low temperatures and at pH ~7.4. Upon administration into the tumor microenvironment—a slightly acidic environment (pH ~ 6.5–6.8)—the carboxyl groups partially protonate. This, combined with body temperature (37 °C), triggers the rapid aggregation of the polymer chains and the formation of a stable gel depot directly within the tumor [80].

The main challenges in the development of these ISSs are as follows:

(a) Reproducibility: The degree of carboxylation and the distribution of functional groups along the polymer chain can vary between batches, affecting the critical gel parameters.
(b) Mechanical strength: Pure carboxymethyl agarose gels often possess insufficient mechanical stability for load-bearing applications (e.g., in articular cartilage), necessitating the creation of composites with other polymers or nanoparticles.
(c) Immunogenicity: Like any polysaccharide of natural origin, even purified agarose may contain trace amounts of pyrogens or elicit an immune response, requiring stringent quality control.

Carboxymethyl agarose represents a versatile and promising platform and is particularly valuable for creating systems responsive to a combination of physiological stimuli (temperature, pH, and ionic strength). Its further development is associated with the advancement of modification methods to produce materials with tailored properties and its integration into complex combined delivery systems.

(2) Aminated guar gum (~37 °C) [81]: Aminated guar gum is a modified polysaccharide, a galactomannan extracted from the seeds of the plant *Cyamopsis tetragonoloba*, consisting of a linear mannose backbone with galactose side branches. It is produced by introducing amino groups (–NH_2_) into the structure of natural guar gum. Based on this modified polymer, an intranasal, thermosensitive system in combination with poloxamer 407 and loaded with rivastigmine has been reported. Aminated guar gum, which combines thermosensitive properties at ~32–34 °C (corresponding to the temperature of the nasal cavity) with high mucoadhesion, together with poloxamer that increases the overall viscosity of the system, represents an optimal platform for intranasal delivery to bypass the blood–brain barrier. Upon administration into the nose, the liquid solution rapidly transforms into a gel that adheres firmly to the mucosa. This ensured prolonged release and direct transport of the drug to the brain, resulting in a 3-fold increase in CNS bioavailability in rats compared to oral administration and a significant improvement in cognitive function in behavioral tests [82].

The main challenges in the development of these ISSs are as follows:

(a) Controlling the degree of amination: Strict control over the modification process is required to ensure reproducible thermosensitive and adhesive properties as even minor variations in the degree of substitution (DS) can significantly affect the LCST.
(b) Viscosity characteristics: The high molecular weight and branched structure of guar gum can result in overly viscous solutions even at low concentrations, complicating injection or intranasal administration. This issue is addressed by partial hydrolysis or the use of copolymers.
(c) Immunogenic potential: As with any polysaccharide of natural origin, there is a theoretical risk of an immune response, necessitating thorough purification from endotoxins and protein impurities.

Aminated guar gum exemplifies a successful case where a relatively simple chemical modification of a classic polymeric excipient can confer the properties of a “smart” biopolymer, combining thermosensitivity, enhanced bioadhesion, and novel biopharmaceutical functions. Its future development is likely to lie in the development of more complex copolymer systems and targeted conjugates to address the challenges of localized and prolonged drug delivery.

(3) Methacrylated hydroxypropyl cellulose (HPC-MA, LCST ~25–38 °C) [83]: Methacrylated hydroxypropyl cellulose is a cellulose derivative modified via the esterification of a portion of its hydroxyl groups with methacrylate functional groups. The base polymer, hydroxypropyl cellulose (HPC), is a semi-synthetic, cold-water-soluble polymer exhibiting thermoreversible gelation with an upper critical solution temperature (UCST) of around 40–45 °C, which is above the physiological range. The introduction of methacrylate groups, typically through a reaction with methacrylic anhydride or methacryloyl chloride, fundamentally alters its properties, conferring the photo- or thermo-initiated polymerization ability and allowing for fine-tuning of the phase transition temperature.

A known combined thermo- and photosensitive system is based on methacrylated hydroxypropyl cellulose for ophthalmic delivery. An HPC-MA solution with an LCST tuned to 33–34 °C thickens upon instillation into the eye due to the thermal effect, increasing contact time. Subsequent mild natural UV exposure at safe doses (or the use of visible-light-activated photoinitiators) induces gentle crosslinking, forming a more stable gel film on the cornea. Such a system for the delivery of cyclosporine A in dry eye syndrome demonstrated an increase in retention time up to 24 h and significant improvement in clinical indicators in preclinical studies [83].

The main challenges in the development of these ISSs are as follows:

(a) Cytotoxicity of photoinitiators: A key limitation is the potential toxicity of traditional UV initiators (e.g., Irgacure 2959) and their degradation products. Active research is focused on developing visible-light-activated initiators and systems with extremely low initiator concentrations [84].
(b) Control of polymerization depth: When using UV light, the penetration depth is limited, which complicates the curing of large volumes. A solution lies in the combination of UV light with thermal initiation or using two-step processes (thermogellation followed by surface photocrosslinking).
(c) Effect of the degree of substitution on properties: Strict control over the methacrylation process is necessary as the degree of substitution directly affects the polymerization rate, the final mechanical properties of the hydrogel, and its compatibility with living cells.

Methacrylated HPC serves as a prominent example of an “engineered” biopolymer, the properties of which can be precisely programmed for a specific biomedical application—from the fabrication of additively manufactured tissue-engineered constructs to “smart” in situ-forming drug depots. Its future is associated with the development of safer initiation systems and the creation of multifunctional composites.

(4) 2-Hydroxy-3-butoxypropylhydroxyethylcellulose (~17–43 °C) [85]: 2-Hydroxy-3-butoxypropylhydroxyethyl cellulose (HBPHEC) is a complex cellulose derivative obtained through the sequential introduction of two types of substituents: hydroxyethyl groups (imparting water solubility and preventing crystallization of the cellulose backbone) and 2-hydroxy-3-butoxypropyl groups. The latter are key contributors to the polymer’s thermosensitive properties. This synthesis is typically conducted in two stages: first, hydroxylethyl cellulose (HEC) is produced, which is then modified with butyl glycidyl ether in an alkaline medium.

The introduction of 2-hydroxy-3-butoxypropyl groups imparts amphiphilic properties to the polymer. The hydroxyethyl fragments and hydroxyl groups remain hydrophilic, while the butyl chains (–C_4_H_9_) create pronounced hydrophobic domains. The balance between these components, regulated by the degree of substitution (DS), determines the phase transition temperature. The LCST of HBPHEC can vary across an exceptionally wide range—approximately from 17 °C to 43 °C—enabling the precise “tuning” of the polymer for a specific physiological or technological application [85,86]. This represents one of the broadest tuning windows among known thermosensitive polymers.

Due to the ability to precisely tune the LCST to 37 °C, HBPHEC is of interest for creating injectable depots. An aqueous polymer solution with an LCST of 35–36 °C, loaded with a model protein (bovine serum albumin, BSA), rapidly formed an in situ gel upon subcutaneous injection in mice. This gel provided a slow, controlled release of the protein over 10–14 days, demonstrating a linear release profile and preservation of the protein’s native structure [87]. This indicates potential for the delivery of peptides, vaccines, and other biopharmaceuticals.

The main challenges in the development of these ISSs are as follows:

(a) Synthesis complexity and cost: The multi-stage synthesis and the necessity for strict control over the degree of substitution for both types of groups render the polymer relatively expensive and challenging to standardize for industrial-scale production.
(b) Regulatory status: HBPHEC is a novel synthetic excipient and lacks an established regulatory dossier in pharmacopeias, unlike its predecessors (HEC and HPC). This requires the developer to conduct a complete package of toxicological and pharmacokinetic studies.
(c) Mechanical strength: Physical gels based on HBPHEC typically possess moderate mechanical strength and may be subject to syneresis (liquid expulsion) during long-term storage, which limits their application in certain implantable systems [88].

Despite these challenges, 2-hydroxy-3-butoxypropylhydroxyethylcellulose represents an outstanding example of how a directed molecular design based on a natural polymer can lead to a material with a uniquely wide and tunable range of properties. Its potential is best realized in applications requiring fine temperature control within the physiological range.

### 3.8. Thermogels Based on Polylactic-co-Glycolic Acid

Another promising thermosensitive polymer is the block copolymer of polylactic-co-glycolic acid (PLGA), or rather its copolymer with polyethylene glycol (PLGA–PEG). PLGA itself is a linear aliphatic copolymer obtained via open-ring block copolymerization of its monomers, lactic acid (LA), and glycolic acid (GA), taken in various ratios. It can be synthesized with any ratio of LA and GA, and its molecular weight (Mw) ranges widely, from less than 10,000 to 200,000 g/mol.

An extensive literature search was conducted to study experiments on the modification of PLGA polymer to obtain thermoreversible drug delivery systems. In 2001, an international group of scientists led by Gaylen M. Zentner synthesized a block copolymer of polylactic-co-glycolic acid (PLGA) and polyethylene glycol (PEG) for the first time [89].

The final polymer with this modification allows for the creation of drug delivery systems in the form of hydrogels, micelles, and nanoparticles, which can include both hydrophobic and hydrophilic drugs. Also, unlike pure PLGA, PLGA–PEG becomes soluble in water and acquires thermoreversible properties, i.e., the modified polymer is biodegradable, phase-dependent, and thermosensitive, which allows for better control of drug release at the site of application [60].

The PLGA–PEG block copolymer is biocompatible, biodegradable, hypoallergenic, and non-toxic to the body. This polymer has also attracted researchers due to its relatively simple and easily controlled synthesis technology, which allows for the variation in the properties (rate of degradation in the body, hydrophilic–lipophilic balance, etc.) of the final polymer [90].

The polyethylene glycol links in the copolymer increase its hydrophilicity and allow it to dissolve in both organic solvents and water while maintaining the integrity of the copolymer in aqueous solutions, unlike the PLGA copolymer. Also, depending on the ratio of lactide and glycolide in the PLGA units and the mass fraction of PEG in the final copolymer, the copolymer of polylactic-co-glycolic acid with polyethylene glycol can respond to different stimuli in the composition of in situ delivery systems.

PLGA–PEG–PLGA block copolymers with a molecular weight of up to 7000 Da and a mass fraction of PEG of up to 30% are of great interest as they have good water solubility and can be used to obtain thermoreversible in situ systems with an LCST ranging from 27 to 37 °C at working concentrations in aqueous solution from 15 to 25%. It is important to note that many studies by different authors demonstrate similar experimental results. The PLGA–PEG–PLGA polymer, which exhibits the parameters required in pharmaceutical development (solubility in water, gel formation at a temperature of 27–37 °C, and an excipient concentration in an aqueous solution of 15 to 25%), contains PLGA units with a molecular weight of 1400 Da to 1700 Da and a PEG unit with a molecular weight of 1500 Da and is a promising candidate for pharmaceutical development [37,91,92,93].

The mechanism of thermally dependent gel formation in aqueous solutions of PLGA–PEG–PLGA is due to the amphiphilic nature of this polymer. Due to the hydrophilic fragments of glycolide and polyethylene glycol, PLGA–PEG–PLGA forms hydrogen bonds with a polar solvent (water). Thus, in solution, it exists in the form of solvated molecules. As the temperature rises and the hydrogen bonds break, the solvates are then destroyed, and the polymer molecules concentrate. A hydrophobic interaction occurs between the lactide fragments of PLGA–PEG when the critical micelle concentration (CMC) is reached. During this interaction, polymer micelles are formed, and, with a further increase in temperature up to the critical gelation temperature (CGT), the micelles form more densely packed structures. Upon reaching a certain threshold concentration, the micelles form gels with different microheterogeneous structures [94].

**Figure 4:**
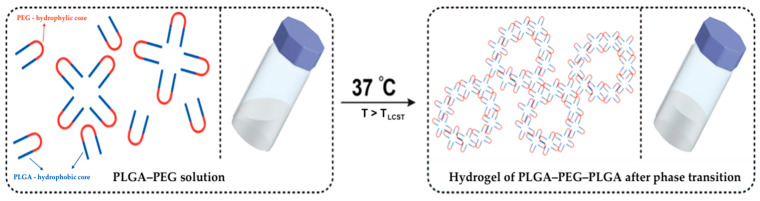
Thermosensitive transition of a PLGA–PEG–PLGA aqueous solution at 37 °C.

Figure 4 illustrates the thermosensitive transition of a PLGA–PEG–PLGA aqueous solution at 37 °C.

The main challenges in the development of these ISSs are as follows:

(1) Complexity of synthesis with reproducible characteristics: Producing a triblock copolymer with a narrow molecular weight distribution (Ð < 1.3), a defined LA/GA ratio in the PLGA blocks, and a precise PEG block length is a non-trivial task. The most common method is ring-opening polymerization of lactide and glycolide in the presence of dihydroxylated PEG as a macroinitiator. Strict control over monomer purity, catalyst activity, and process conditions is necessary to avoid the formation of diblocks, homopolymers, or cyclic oligomers [91].
(2) Hydrolysis sensitivity and degradation control: While the hydrolysis of ester bonds in PLGA is desirable for biodegradability, it begins as soon as the polymer is dissolved in water to prepare the formulation. The accumulation of acidic degradation products (lactic and glycolic acids) within the gel depot can lead to an autocatalytic effect: local pH reduction, accelerated polymer breakdown, denaturation of sensitive active substances (proteins), and a potential inflammatory tissue response [95]. Mitigation strategies include buffering the formulation, using polymers with higher content of glycolide (which degrades faster and reduces the drop in pH), or incorporating basic agents.
(3) Sterilization challenges of the final dosage form: Like other thermosensitive systems, the finished solution or gel cannot be sterilized by heat. The primary methods are aseptic manufacturing (most common but expensive) or sterilization of the finished lyophilized polymer powder by gamma irradiation, followed by dissolution in a sterile solvent [96].

The PLGA–PEG–PLGA triblock copolymer remains the “gold standard” among synthetic injectable thermoreversible systems due to its predictable properties and extensive research foundation. Its future is tied to the development of more complex multiblock or star-shaped architectures for better control over rheology and degradation, as well as the creation of combined systems where the gel serves as a matrix for immobilizing targeting ligands or nanoparticles. Despite regulatory and technological challenges, this polymer class holds high potential for clinical translation, particularly for niche applications requiring prolonged and localized delivery of high-value biopharmaceuticals.

### 3.9. Polyethylene Glycol-Based Thermogels

Numerous polyethylene glycol-based monomers have been described in the literature, and the main representatives of this class are copolymers of polyethylene oxide (PEO) and polypropylene oxide (PPO) with an LCST in the range from 20 °C to 85 °C, containing ethylene glycol units in the main chain and commercially available under the names Pluronics^®^ and Tetronics^®^ [21,97].

The amphiphilic balance in the structure of oligethylene oxides is the main reason for the thermosensitive properties of these types of polymers. Copolymers based on monomers consisting of methacrylates crosslinked with five or more ethylene glycol units in the side chain capable of polymerization are known. However, the LCST of ethylene glycol polymers is typically in the range of 80–100 °C, which limits their use in drug delivery systems [98,99].

Nevertheless, such polymer matrices continue to be actively researched due to their high biocompatibility and low toxicity.

Methoxy-terminated oligo(ethylene glycol) (OEG) links are sometimes preferred because they exhibit little or no hysteresis upon cooling. At the same time, hydroxy-terminated OEG links have the advantage of the presence of an OH group at the end of the chain, which can be modified to tune the LCST. Polymers with carbon–carbon backbones (e.g., acrylates, methacrylates, and styrenes) with non-extended ethylene glycol side chains (e.g., mono-, di-, and tri-ethylene glycols) typically exhibit lower LCST values in aqueous solution compared to polymers with long ethylene glycol side chains (i.e., oligethylene glycols). The hydrophobicity of the polymer increases as the length of the ethylene glycol units decreases due to the increase in the overall proportion of the hydrocarbon backbone. For example, poly-(methylethylene glycol)2-methacrylate (PMEO_2_MA) with two ethylene glycol units and poly-(methylethylene glycol)3-methacrylate (PMEO_3_MA) with three ethylene glycol units have an LCST in the range of about 26 °C and 52 °C, respectively. For a more precise adjustment of the TPC, the copolymerization of short and long (meth)acrylates of ethylene glycol can be carried out with various monomer ratios [100,101,102].

Polymers can contain ethylene glycol units in the side chain with various main chains, including polymethacrylate (PEOMA), polyacrylate (PEOA), polystyrene (PEOSt), polyvinyl ether (PEGVE), polynorbornene (PEGNB), polylactide (PEOLA), and polyphosphazene (PBEEP). Early studies of thermosensitive polymers mainly focused on PNIPAM and PEOMA derivatives. However, a wide range of other polymers also demonstrate promising thermosensitive behavior in aqueous solution. For example, poly-N-vinylcaprolactam (PNVCL) and polyoxazolines exhibit LCST behavior. Similarly to PNIPAM, PNVCL is hydrophilic and soluble in water at room temperature and below. PNVCL exhibits behavior characteristic of these polymers, i.e., when an aqueous solution of this polymer is heated, a solution–gel transition occurs according to the mechanism described above in the temperature range from 25 °C to 35 °C [103,104,105,106].

There are also studies in which various biopolymers are copolymerized with polyethylene glycols and methoxypolyethylene glycols in order to impart thermosensitive properties to compounds such as cyclodextrin, enzymes, proteins, or oligonucleotides [107,108,109].

The main challenges in the development of these ISSs are as follows:

(1) High cost and complexity of synthesizing tunable copolymers: While poloxamers are commercially available and affordable, the synthesis of POEGMA or other complex PEG-containing copolymers with controlled architecture and low polydispersity requires expensive catalysts, highly purified monomers, and sophisticated polymerization techniques (RAFT or ATRP), significantly increasing the cost of the final material [110].
(2) Difficulty in controlling the LCST for complex compositions: The LCST of POEGMA-based systems is influenced by numerous factors: backbone length, length, and density of the OEG side chains, the nature of the OEG end group (methoxy or hydroxy), and the presence of comonomers. Minor variations in synthesis can lead to significant shifts in the gelation temperature, necessitating rigorous quality control for each batch [111].
(3) Limited mechanical strength of physical gels: Gels formed through physical interactions (poloxamers and some POEGMAs) often possess a low elastic modulus and are prone to syneresis (liquid expulsion) or rapid erosion in physiological environments due to the fact that physical gels are held together by relatively weak, transient forces like hydrogen bonding, hydrophobic interactions, or molecular entanglement. This limits their application for creating long-term implantable depots [17].
(4) Sterilization and stability issues: PEG-containing polymers can undergo oxidative degradation during sterilization via gamma irradiation or autoclaving. In thermosensitive systems, filtration sterilization is often the only viable option, which imposes constraints on the viscosity of the initial solution [112].

Thus, the class of PEG-containing thermogels demonstrates an evolution from simple yet effective systems to sophisticated, “intelligent” materials. Their successful clinical translation will depend on the ability to overcome the critical bottleneck between the high cost and complexity of developing novel polymers and the necessity to provide compelling evidence of their clinical utility.

#### 3.9.1. Poly(N-Vinylcaprolactam) (PNVCL)

PNVCL (poly(N-vinylcaprolactam)) is a non-toxic, biocompatible, and non-immunogenic polymer with a lower critical solution temperature (LCST) in the physiologically relevant range of 25–35 °C, making it a promising alternative to the widely studied poly-N-isopropylacrylamide (PNIPAAm) [113].

A key advantage of PNVCL is that it does not form toxic low-molecular-weight amines (such as ammonia) during hydrolysis, significantly enhancing its safety profile for in vivo applications [114]. The thermosensitive properties of PNVCL arise from the balance between the hydration of its amide groups and the hydrophobic interactions of its cyclic caprolactam moiety. The phase transition temperature can be readily adjusted via copolymerization with hydrophilic (e.g., vinylpyrrolidone) or hydrophobic monomers or by forming interpolymer complexes. Hydrogels based on PNVCL exhibit high swelling capacity and the capacity for a controlled release of both hydrophilic and lipophilic compounds.

Promising application areas include ophthalmic in situ gels for the treatment of glaucoma and keratitis, transdermal systems for prolonged analgesic delivery, and localized intratumoral therapy. One of the most promising directions is the development of long-acting eye drops. Traditional aqueous solutions are rapidly cleared via the nasolacrimal duct, resulting in low bioavailability (<5%). Thermosensitive PNVCL-based formulations, which are liquid at room temperature (~25 °C), rapidly gel upon instillation into the conjunctival sac (temperature ~34 °C), increasing corneal contact time. For example, an in situ gel based on a copolymer of PNVCL and polyvinylpyrrolidone (PVP) for delivering the anti-glaucoma drug timolol maleate demonstrated a 4–5-fold increase in corneal retention time in rabbits compared to a standard solution and provided controlled release over 12 h. Similar systems are being studied for delivering antibiotics (e.g., ciprofloxacin) in the treatment of bacterial keratitis [115].

The reversible gelation ability near body temperature opens prospects for creating injectable depots directly within tumor tissue. Experimental studies have shown that a solution of a PNVCL–polyethylene glycol (PEG) copolymer containing the cytostatic drug doxorubicin forms a stable local depot upon injection into a mouse tumor (B16F10 melanoma). The gel matrix provides slow drug release over 10–14 days, maintaining high local concentrations and minimizing systemic toxicity, leading to significant tumor growth inhibition compared to an intravenous administration of the same drug [116,117].

Despite the potential of PNVCL, the implementation of PNVCL systems is hindered by technological challenges. Obtaining polymers with reproducible characteristics (narrow molecular weight distribution and target LCST) requires strict control over radical polymerization conditions. The final product cost can be reduced by developing efficient methods for purifying residual monomers and utilizing copolymerization with less expensive components (e.g., vinylpyrrolidone). An important aspect for pharmaceutical application is also studying the long-term stability of PNVCL-based formulations during storage and their compatibility with sterilization methods [114].

To reduce risks and development costs, the following are recommended: (1) using PNVCL in copolymers with already approved polymers (e.g., PEG or PVP), where PNVCL constitutes a smaller fraction; (2) applying a Quality by Design (QbD) strategy from the earliest stages to establish correlations between polymer parameters and critical product qualities; and (3) publishing preclinical data in peer-reviewed journals to build an evidence base and reduce regulatory uncertainty for the entire scientific community.

Thus, PNVCL represents a versatile and promising polymeric platform, with research actively transitioning from fundamental property studies to solving specific applied problems in controlled drug delivery and regenerative medicine. Further progress in this field will depend on successfully addressing the technological challenges of scaling up and standardizing the production of this polymer.

#### 3.9.2. Poly(2-Oxazoline)s (POxs)

Poly(2-oxazoline)s, particularly poly(2-methyl-2-oxazoline) (PMeOx), poly(2-ethyl-2-oxazoline) (PEtOx), and poly(2-propyl-2-oxazoline) (PPropOx), represent highly biocompatible polymers with a tunable LCST (range 25–65 °C) regulated by the length of the side alkyl chain. Due to the presence of polar amide bonds in the backbone, analogous to peptide bonds, they are often referred to as “pseudo-polypeptides”. POxs exhibit a pronounced “stealth effect” in vivo, effectively reducing opsonization by macrophages and increasing circulation time, which aligns them with polyethylene glycol (PEG). This polymer class is distinguished by high stability and chemical versatility: the presence of active terminal groups allows for directed conjugate synthesis with drugs or targeting ligands [118].

Owing to the combination of stealth properties, biocompatibility, and the ability to precisely tune physicochemical characteristics, poly(2-oxazoline)s are considered among the most promising platforms for the next generation of therapeutic systems. Their application extends beyond simple thermosensitive gels to encompass the creation of highly functional nanoconstructs and “smart” polymeric therapeutics [119].

POx triblock copolymers, such as PEtOx-b-PPropOx-b-PEtOx, are capable of forming transparent, stable, and biodegradable gels at body temperature in the concentration range of 10–25% *w*/*v*. Such systems are being investigated as alternatives to poloxamers for creating injectable depots. For example, a POx-based hydrogel containing a somatostatin analog (octreotide) provided linear peptide release over 14 days in vitro, demonstrating potential for treating acromegaly without the need for frequent injections. The biodegradation of such gels occurs via the hydrolysis of the backbone amide bonds into non-toxic oligomers, distinguishing them from non-degradable poloxamers [120].

Polymers with dual responsiveness can be created through the copolymerization of 2-oxazolines with monomers bearing functional groups. For instance, introducing monomers with carboxyl groups into the side chain enables the production of pH- and thermosensitive systems for oral delivery. Such copolymers remain in a collapsed state in the acidic gastric environment, protecting the encapsulated drug (e.g., insulin or peptide-based antidiabetic agents), and unfold to form a gel in the alkaline environment of the small intestine, promoting adsorption [121].

Incorporating disulfide bridges into the structure creates redox-sensitive POxs that destabilize in the reductive environment of the cell cytoplasm, enabling intracellular release [122].

The primary operational advantage of POxs is the flexibility and controllability of their synthesis via living cationic polymerization. This enables the following:

(1) Precise control of molecular weight (Ð < 1.1).
(2) Synthesis of block copolymers with defined architecture (diblocks, triblocks, graft copolymers).
(3) Introduction of a wide range of functional groups (amines, carboxylic acids, azides, alkynes) at chain ends or side groups for subsequent conjugation [123].

Thus, POxs represent not merely a new material but a universal modular platform for constructing task-specific delivery systems. Their potential extends from creating simple injectable gels to developing complex theranostic (therapeutic diagnostic) nanocomplexes that combine prolonged circulation, controlled release, and specific targeting. Despite patent and production challenges, POxs are rightfully considered among the primary candidates to replace PEG in next-generation delivery technologies.

#### 3.9.3. Elastin-like Polypeptides (ELPs)

Elastin-like polypeptides are bioinspired recombinant protein polymers composed of repeating pentapeptide motifs (Val-Pro-Gly-X-Gly, where X is any “guest” amino acid except proline). Their uniqueness lies in their ability to undergo a reversible, temperature-induced solution–hydrogel phase transition within a strictly defined temperature range (typically 30–60 °C). The LCST of ELPs can be programmed with exceptional precision at the genetic level by modifying (1) the nature of the “guest” amino acid X (increased hydrophobicity lowers the LCST); (2) the length of the polypeptide chain; and (3) the number and sequence of repeating blocks. ELPs are biodegradable into non-toxic amino acids, exhibit extremely low immunogenicity, and possess a natural ability to target tumor tissue via the enhanced permeability and retention (EPR) effect [124].

The unique properties of ELPs—biocompatibility, precise tunability, and reversible aggregation—create opportunities for developing highly specific delivery systems that can be “programmed” to address biomedical challenges.

The most advanced direction is the creation of thermosensitive conjugates for oncology. A classic example is a doxorubicin (DOX) conjugate with ELPs (DOX–ELP). The conjugate is administered systemically as a solution below the polypeptide’s LCST. Due to the EPR effect, it accumulates in the tumor, where the local temperature is often slightly higher (by 1–2 °C) than in healthy tissues. Upon reaching its LCST in the tumor interstitium, the ELP conjugate undergoes reversible aggregation, forming a biodegradable depot that provides prolonged (over several days or weeks) release of the active substance directly at the target site. This strategy overcomes the main drawbacks of free doxorubicin: systemic toxicity (cardiotoxicity) and rapid clearance. In preclinical studies on models of pancreatic cancer and triple-negative breast cancer, this approach demonstrated a significant increase in the therapeutic index and suppression of tumor growth with reduced side effects [125].

The protein–peptide nature of ELPs makes them ideal carriers for therapeutic biomolecules, which often require gentle encapsulation conditions. Using genetic engineering, recombinant chimeric proteins are created where a therapeutic peptide or protein (e.g., cytokine, growth factor) is directly fused to an ELP sequence at the genetic level. Such chimeras retain the activity of the biomolecule, while the ELP domain provides prolonged circulation and thermosensitivity. For example, a chimeric protein based on ELPs and the immunostimulatory cytokine interleukin-2 (IL-2) forms a depot upon local injection into a tumor, overcoming the systemic toxicity of high-dose IL-2 and activating an anti-tumor immune response directly within the tumor microenvironment [126].

In the delivery of nucleic acids (siRNA, DNA), ELPs are cationized by incorporating lysine or arginine residues into their sequence. The resulting ELP polyplexes effectively condense nucleic acids, protect them from degradation, and, due to their thermal response, can release the cargo inside the cell upon a temperature change. Such systems are being investigated for gene therapy and genome editing [127].

The typical architecture of a recombinant ELP conjugate for delivery comprises several domains: [Therapeutic agent]—[Peptide linker (enzyme- or pH-sensitive)]—[ELP sequence (defines LCST)]—[Targeting peptide ligand (optional)].

The flexibility of genetic design enables the independent optimization of each domain for a specific therapeutic objective.

Thus, ELPs represent not just another class of carrier polymers but a programmable technology platform that can elevate drug delivery to a qualitatively new level of precision and controllability, fully harnessing the biological characteristics of the pathological site.

#### 3.9.4. Poly(N-Vinylamides): Polyvinylpyrrolidone (PVP) and Its Derivatives

While polyvinylpyrrolidone (PVP) is historically known as a stabilizer and carrier lacking intrinsic thermosensitivity, its copolymers are of significant interest. The copolymerization of N-vinylpyrrolidone with more hydrophobic monomers, such as N-vinylcaprolactam or alkyl acrylates, enables the production of materials with a tunable LCST. Such copolymers combine high biocompatibility—proven through decades of clinical use of PVP as a plasma expander—with the ability to fine-tune the gelation temperature. Their key advantages are exceptional solubility in water and physiological media, chemical stability, and good compatibility with a wide range of drugs, including poorly soluble compounds. Thermosensitive gels based on PVP copolymers are being investigated for their ability to create injectable depots for antibiotics and local anesthetics and as coatings for medical implants with controlled release [128].

Copolymers of N-vinylpyrrolidone with N-vinylcaprolactam or alkyl methacrylates demonstrate a clearly adjustable LCST in the range of 30–38 °C. For example, a PVP-co-NVCL copolymer (70:30 ratio) with an LCST of around 34 °C is used to create injectable gels containing vancomycin. A solution administered subcutaneously at room temperature rapidly forms a gel in the tissue environment, maintaining a local antibiotic concentration above the MIC for 7–10 days, which is effective for treating resistant skin infections without systemic toxicity. Similar systems are being studied for delivering anticancer drugs (such as cisplatin) directly to tumor sites [129].

The high solubility and stability of PVP across the wide pH range of the gastrointestinal tract allow for its use in creating complex formulations. For instance, thermoreversible gels based on a copolymer of PVP with polycaprolactone (PCL) are being developed for gastroprotective delivery of non-steroidal anti-inflammatory drugs (NSAIDs). The system remains in a gel state in the stomach (37 °C), protecting the mucosa from the drug’s irritant effects, and then dissolves in the intestine, ensuring complete release. Furthermore, PVP is widely used in hot-melt extrusion technology for producing amorphous solid dispersions, significantly enhancing the bioavailability of poorly soluble drugs (e.g., itraconazole and ritonavir) [130].

A key feature of PVP-containing systems is their processing-friendly nature:

(1) Broad compatibility with most pharmacopeia-included solvents and active substances;
(2) Stability under thermal processing (e.g., during sterilization);
(3) Usability in various manufacturing processes (spray drying, extrusion, and lyophilization);
(4) Ease of property modification through copolymerization.

However, when working with PVP, its hygroscopic nature and ability to form complexes with certain substances (e.g., polyphenols) must be taken into account as these properties can influence release kinetics [131].

Thus, PVP and its derivatives, particularly thermosensitive copolymers, constitute a foundation for creating intelligent delivery systems with spatial and temporal control. Their further development is associated with the design of more complex copolymer architectures (star-shaped and graft copolymers) and combinations with other functional polymers to address specific targeted delivery challenges.

A comparative table of polymers for creating ISSs is presented below (Table 1).

There exists a wide spectrum of polymeric platforms, each occupying its own niche depending on the requirements of a specific application. The selection is not limited to classical systems (poloxamers or chitosan/β-GP). An arsenal of highly engineered materials (POxs, ELPs, tunable POEGMAs, and modified polysaccharides) has emerged, the properties of which can be precisely programmed at the molecular level.

The classic “simplicity vs. functionality” trade-off remains valid. Systems that are simplest to use and have a history of safe application (e.g., poloxamers) often lag behind more complex analogs in parameters such as mechanical strength, duration of action, biodegradability, or targeting precision. Conversely, advanced platforms (e.g., ELPs and POxs) offer unprecedented control, but their development is associated with high cost, complex synthesis, and a lengthy regulatory pathway.

Natural and bio-derived polymers (chitosan, xyloglucan, and agarose) hold particular value for mucosal and topical applications, where bioadhesion, biocompatibility, and natural origin are critically important. However, their main barrier is the high variability in their properties, necessitating the strictest control over the raw material source and modification process.

The key technological challenges common to most new systems are as follows:

(1) Synthesis reproducibility and standardization (particularly for block copolymers and modified biopolymers).
(2) Control over the biodegradation process and the impact of degradation products (e.g., acidification for PLGA and immune response to PEG).
(3) Development of sterilization methods that do not compromise thermosensitive properties.
(4) Demonstration of long-term safety and absence of immunogenicity.

Regulatory status is a determining factor for development strategy. Utilizing already-approved excipients (poloxamer or chitosan for wounds) significantly accelerates the preclinical stages. Any new copolymer or conjugate (PEG–chitosan, POx, or a specific PLGA–PEG–PLGA triblock) is considered a new chemical entity, requiring a full registration dossier, which drastically increases the time and cost of bringing a product to market.

The strategic selection of a matrix should be based on a clear definition of the objective:

(1) For short-term sustained action (1–3 days) and mucosal systems, poloxamers or chitosan/β-GP may be optimal.
(2) For long-term injectable depots (weeks or months) and delivery of biopharmaceuticals, biodegradable copolymers (PLGA–PEG–PLGA) or POxs are promising.
(3) For tasks requiring high-precision targeting (e.g., in oncology) or tissue engineering, programmable platforms are most suitable: ELPs or functionalized POEGMA.
(4) For topical applications and cosmeceuticals, modified polysaccharides (carboxylated agarose, aminated guar) are effective.

The future development of the field is linked to the trend toward hybridization and the creation of multifunctional systems. Combining the advantages of different classes (e.g., the biological function of ELPs or chitosan with the controlled architecture and “stealth properties” of POxs) will enable the creation of next-generation systems that overcome the limitations of individual materials. An additional powerful trend is the integration of thermosensitive hydrogels with additive manufacturing technologies (3D bioprinting) for creating personalized implants and delivery systems.

Thus, the contemporary researcher possesses an extensive and differentiated toolkit of thermosensitive polymers. The success of development will be determined not so much by the search for a “universal” polymer, but by the ability to make a rational selection that accounts for a multifaceted set of requirements: from the biological objective and route of administration to economic and regulatory constraints, as well as the readiness to address the accompanying technological and analytical challenges.

## 4. Integrative Analysis and Future Perspectives

One of the reasons for the low success rate of pharmaceutical development is the lack of clear structured information about the capabilities and shortcomings of known thermosensitive polymers, compiled on the basis of a broad pool of research. Despite the similar mechanism of thermally dependent phase transition, the polymers discussed above differ significantly from each other in terms of parameters that play an important role in the quality of a drug. Taking these polymer characteristics into account will help researchers make informed choices about composition and increase the success rate of pharmaceutical development.

### 4.1. Thermoreversible and Thermosensitive Irreversible Gelation

Polymers that undergo a phase transition when the temperature changes are often called “reversible” polymers because, when the temperature subsequently rises and exceeds the gelation range, they undergo a reverse gel–sol phase transition while retaining the original viscosity of the solution [133]. Such polymers include PEO and PPO block copolymers (pluronics), PLGA–PEG–PLGA, and many other synthetic polymers discussed above. Thus, thermoreversible polymers with an LCST and a UCST are used in both the sol–gel transition and the reverse gel–sol transition [149]. However, not all thermosensitive polymers are thermoreversible. A striking example of a thermosensitive but not thermoreversible system is the chitosan/BGF system, which, after a temperature-dependent transition and subsequent cooling, exhibits the behavior of an unstable suspension because of the precipitation of chitosan.

Thermoresponsive ISSs are the most versatile delivery systems due to the constant temperature difference between drug storage and in vivo. However, studies show that it is often difficult to maintain the ISS designed for the experiment phase transition temperature in long-term storage. Some researchers believe that a phase transition occurring at a temperature that exactly matches the temperature of the application site is not a prerequisite for a high-quality and effective thermosensitive ISS. The introduction of the system at a temperature of 4–5 °C, as implemented in some published studies, ensures the desired low viscosity during administration and the fastest possible gel formation upon contact with the mucous membrane, due to a sharp jump in the temperature of the ISS by almost 30 °C.

**Figure 5:**
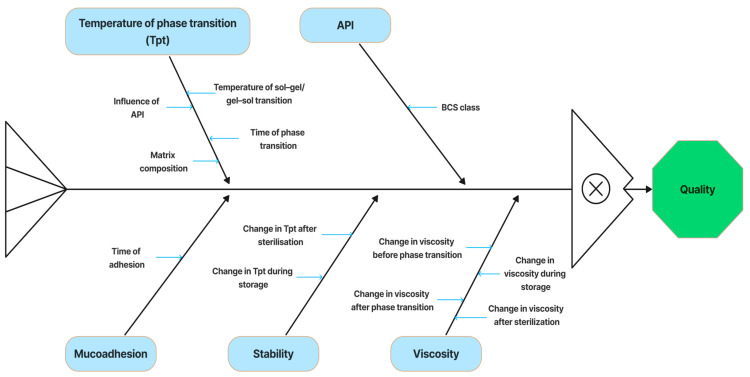
Risk analysis in the development of thermosensitive ISSs.

Given the risks of this approach, such as insufficient viscosity of the composition at the temperature of administration and delayed gel formation during application, it should be noted that, from a biopharmaceutical point of view, the administration of such highly cooled drugs to patients does not always result in sufficient compliance and, in some cases, may even be unacceptable. In addition, changing the gel formation temperature specified in the design significantly reduces or even eliminates the targeting of ISSs. Studies conducted on the physicochemical properties of the main thermosensitive polymers, as well as an analysis of the literature, have made it possible to formulate and rank the risks arising in the development of thermosensitive ISSs (Figure 5).

The phase transition temperature is influenced by a number of factors: the composition of the polymer matrix, more specifically the ratio of hydrophilic and hydrophobic blocks; the introduction of additional excipients; and the type and concentration of the API. Other important parameters for a high-quality and effective thermosensitive ISS are the gelation rate (phase transition time) and the characteristics of the reverse gel–sol transition.

The introduction of additional additives must be scientifically justified since exceeding a certain concentration can lead to a decrease or even complete loss of the system’s thermosensitivity. Thus, it is advisable to use proven combinations of polymers with proven optimal characteristics. Figure 5 illustrates risk analysis in the development of ISSs.

Creating a database of such combinations (“thermosensitive matrix database”) could significantly accelerate the process of developing drugs based on thermosensitive ISSs.

It is important to note that the introduction of the API can also significantly change the parameters of the ISS. In our own study (unpublished work) we studied the effect of adding the API (ribavirin) on the rheological properties of a thermosensitive ISS composed of 16% poloxamer 407 and 2% chitosan formiate [135].

**Figure 6:**
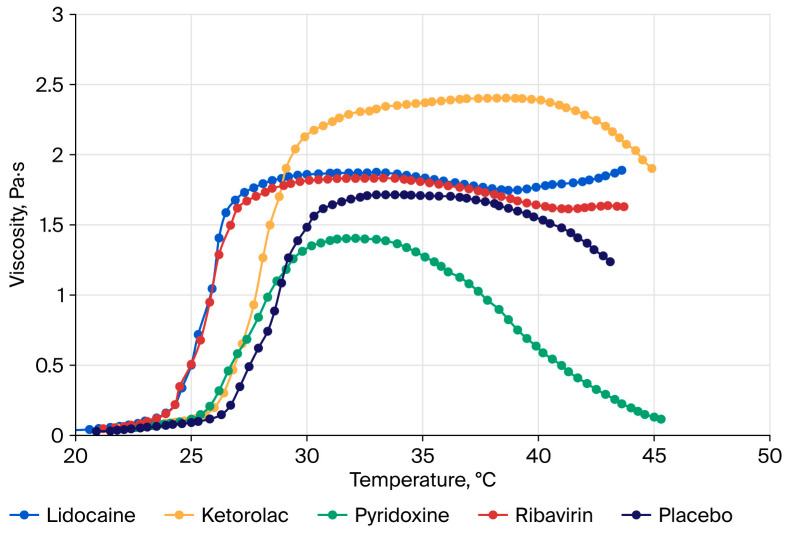
Viscosity curves for samples based on poloxamer 407 with and without BCS class I and III APIs (BCS class I—lidocaine, ketorolac; BCS class III—pyridoxine, ribavirine).

It can be seen on the averaged rheograms (Figure 6) [135] obtained after five consecutive measurements of the systems on a rotational viscometer at a constant shear rate of 100 s^−1^ that the introduction of ribavirin significantly reduces the maximum plastic viscosity of the system (from 0.75 to 0.42 Pa·s) while having a negligible effect on the nature of thermosensitivity. The maximum viscosity (phase transition) of the placebo system and the system with ribavirin is reached at a temperature of about 31 °C. At the same time, the length of the “plateau” zone on the rheogram preceding the reverse gel–sol transition increases after the introduction of the API. Viscosity reversal for the placebo composition occurs at a temperature of 32.1 °C and for the ISS ribavirin at 32.9 °C.

In our own studies [135], we also hypothesized that the gelation temperature of thermosensitive drug delivery systems depends on the solubility and permeability parameters of the active substance. Based on a pool of thermosensitive compositions based on poloxamer 407 and additional ingredients (PEG 1500 and poloxamer 188), it was found that Class I BCS substances with good water solubility and high permeability significantly reduced the gel formation temperature in combinations with poloxamers but had virtually no effect on it in compositions with polyethylene glycol (or had a lesser effect). Class III BCS substances had no effect on the gel formation temperature in combinations of poloxamers and poloxamers with polyethylene glycol while significantly changing the temperature and rheological characteristics of the system with pure poloxamer 407.

Thus, when working with the risk of changing the thermosensitivity characteristics that arise during the development of ISSs, it is not permissible to make an approximate assessment of the indicators based on a pool of placebo compositions since their characteristics will be significantly changed after the final composition is reached and the active pharmaceutical ingredient is introduced.

The temperature-dependent phase transition of ISSs is recorded according to the change in the viscosity of the system. This is why the study of viscosity in particular and rheological characteristics in general is a mandatory step in the development and routine analysis of thermosensitive ISSs. The viscosity of the gel formed in situ or another system (e.g., an implant) affects the kinetics of API release and the duration of the drug’s prolonged effect. Viscosity also often correlates with mucoadhesion parameters, although these characteristics are not always directly dependent on each other [150,151]. However, viscosity undoubtedly plays a significant role in counteracting the natural clearance of the site of administration attached to the mucous membrane ISS. The system may lose its initial viscosity characteristics in both the sol and gel states during storage, as well as after any type of physical impact in the technological process (active dispersion, ultrasonic treatment). It is important to note the possible risk of viscosity loss during the technological process for heat-sensitive ISSs undergoing sterilization.

The mucoadhesion parameter is the second indicator (after the phase transition temperature) that is subject to correction by developers when creating a new ISS. The effective attachment of the system to the site of action on the mucous membrane is a limiting factor in the therapeutic effect of the delivery system. However, the introduction of additional mucoadhesive agents, as in traditional application forms, into stimulus-sensitive ISSs is impossible as this may affect the sensitivity of the delivery system to the selected stimulus. Thus, most researchers are working on a different approach: adding mucoadhesive properties to the sensitive polymer itself, in the case of its targeted synthesis. When screening candidate substances with comparable properties of sensitivity to temperature stimuli, preference is given to the most mucoadhesive of them. Examples of such substances are xyloglucan or chitosan conjugate with 2-iminothiolan, as discussed above.

The risks described above can be managed by following a methodologically sound study design based on reproducible methods for evaluating critical system parameters during pharmaceutical development.

### 4.2. Design of Studies of Drugs Based on Thermosensitive Systems

The main parameter for screening any ISS will be the evidence of the formation of the final form at the site of application, as intended by the design. To this end, researchers use in vitro, ex vivo, and in vivo methods.

Most experimental studies of thermosensitive ISSs determine critical quality attributes (CQAs) such as gel formation temperature, gel strength, and gel formation time. Mucosal adhesion, rheological properties, and API release are also studied.

ISS parameters related to the sol–gel transition are in most cases evaluated in vitro in an artificial fluid environment, the composition of which correlates with the biological environment at the site of administration. For example, an artificial nasal fluid for intranasal systems is prepared that contains electrolytes: 8.77 mg/mL NaCl, 2.98 mg/mL KCl, and 0.59 mg/mL CaCl_2_ [152]; for vaginal systems, an artificial vaginal secretion is prepared with a composition of 0.351% NaCl, 0.140% KOH, 0.022% Ca(OH)_2_, 0.0018% bovine serum albumin, 0.2% lactic acid, 0.1% acetic acid, 0.016% glycerol, 0.04% urea, and 0.5% glucose [153]; for the ophthalmic ISS, an artificial tear-type medium (NaCl 0.67%, NaHCO_3_ 0.20%, and CaCl_2_ × 2H_2_O 0.008% [154]) or physiological solution is employed.

This reproduces the mineral composition and pH of physiological fluid. Tests are carried out in this medium under thermostatic conditions to visually or instrumentally record the sol–gel transition [2,155,156].

Most researchers determine the gelation temperature using the method described by Gilbert et al. in a 1987 article: 2 mL of the prepared composition is transferred to a test tube (10 mL with a diameter of 1.0 cm), sealed with a cap or PARAFILM^®^ film, and thermostatizated at 8 °C in a water bath. The temperature of the water bath is systematically increased in 2 °C increments in the range from 8 °C to 18 °C and then in 1 °C increments every 10 min to establish the gelation temperature. Gelation is visually recorded when the meniscus does not move when the test tube is turned to a horizontal position [132].

Similar methods are also used to determine the gel formation time and gel strength, which are characteristic values for researchers. Gel strength is a characteristic that demonstrates the ability of the composition to overcome mucociliary clearance. In a previous study [157], a sample of each composition was transferred to a 100 mL graduated cylinder, followed by thermostatization in a water bath at 34 °C until gel formation. Then, a 35 g weight was placed on the resulting gel. The strength of the gel, indicating the viscosity of the gel at physiological temperature in the nasal cavity, was determined as the time required for the weight to penetrate the gel to a depth of 5 cm.

The most accurate indicators of the strength of the gels formed, their fluidity and viscosity, are characterized via the rheological method of investigation. Rotational viscometry is used to perform such tests. In [158], a Brookfield DV–II+ viscometer (Brookfield Engineering Laboratories Inc., Middleboro, MA, USA) was used. The measurement was performed by increasing the spindle rotation speed to 100 rpm.

Canadian scientists from McMaster University proposed a simple but effective and reproducible test for the in vitro assessment of the mucoadhesion strength of polymer compositions after in situ gel formation in the nasal cavity [159]. The test polymer composition was sprayed onto two plates with a 2% mucin solution on the surface, which were placed overlapping each other. This method is known among the techniques for determining the spreadability of soft dosage forms and is called “slip and drag” [160]. After in situ gel formation, this structure was placed in a special device that applied tension to the plates in opposite directions with a certain force and relaxation interval. The force of mucoadhesion was calculated by constructing a grading graph. The dependence of the tensile force on the final position of the plates as well as the dependence of the force on the distance by which the plates could be shifted relative to each other were calculated.

Ex vivo methods are also used to assess the strength of mucoadhesion. Thus, in a study by Salem HF et al. [157], intact nasal tissue mucosa from sheep obtained within 1 h after slaughter was used as an ex vivo model to determine the intranasal ISS index. The isolated mucous membrane was cleaned in a 0.9% sodium chloride solution. To study the strength of mucoadhesion, sections of the nasal mucosa selected for the study (2.5 × 1 cm^2^ in size) were fixed on a microscope slide using cyanoacrylate glue, and the force required to separate the tissues with a uniformly distributed in situ gel sample (weighing 1.0 g) between them was determined gravimetrically [2].

Some studies also determine the time of mucoadhesion. The mucosa of freshly slaughtered cattle (ex vivo) is also used in these tests and is pretreated with an artificial fluid, the composition of which correlates with the site of application. The mucosal sections are fixed on a glass cup with the mucosal surface facing outwards. A small amount of the ISS is placed on the surface of the treated mucosa, after which the resulting model is placed in a cell containing 100 mL of artificial biological fluid and slowly stirred at 10 revolutions per minute. The time required for gel erosion in situ was noted visually and considered as the time of in vitro mucoadhesion [161].

From the examples given, most ISS researchers generate original and modify known techniques that bring the research conditions closer to in vivo. At the same time, many of the methods published to date cannot be validated or even reproduced, so the results obtained by different research groups cannot be compared. This occurs due to the fact that unlike tablets or capsules, there are no universally accepted “gold standard” or compendial methods (e.g., specific USP apparatus) for testing ISS. Each research group often develops unique, custom-built adapters or modified dissolution setups to control the shape and surface area of the forming gel, making inter-laboratory comparisons nearly impossible [162].

It is very important to note the peculiarity of measuring mucoadhesion in thermosensitive ISSs. Due to the temperature-dependent increase in viscosity, which, as already mentioned, often correlates with the strength of mucoadhesion, it is advisable to measure mucoadhesion at the physiological or pathological temperature of the application site. Thus, methods that allow for thermostatting will have advantages in analyzing this indicator in thermosensitive ISSs.

The degree of ISS adhesion to the mucosa can also be indirectly assessed using a method developed with the application of rotational viscometry. Rheological analysis is an in vitro method for determining adhesion, which allows the behavior of ISSs to be predicted in vivo and their structural interactions to be investigated by interpreting changes following physicochemical interactions.

The use of instrumental methods for the analysis of thermosensitive ISSs partially solves the issues of reproducibility and validation (as, for example, in the case of determining the phase transition parameter using rotational viscometry), but such methods are not reliably biorelevant. In addition, in vitro experiments often take into account only some of the parameters of the site of application (temperature, pH, and mucin content), and models that include more than two factors of the ISS exposure environment are rare.

It is possible to reproduce the multifactorial nature of the application site using in vivo or ex vivo models for screening. However, due to the multitude of conditions for obtaining, transporting, and storing ex vivo samples, and their inability to be reused, it is impractical to use these techniques at the screening stage of large pools of compositions, both from a financial and ethical point of view.

One possible solution to this problem is the development of in vitro models: standardized devices suitable for reuse, with geometric parameters that replicate the target organ and take into account three or more physiological factors of the site of application.

Today, such models are conditionally divided into rigid, reusable, anatomically correct models created using 3D printing based on published data from studies of the relevant organs, irrigated with artificial bio-relevant fluids, suitable for determining the gelation temperature, distribution after administration, and indicators characterizing mucoadhesion, and soft, disposable models created on the basis of polymer compositions (agar, polyacrylate, hyaluronic acid), which simulate organs or tissues in terms of their physicochemical properties and are suitable for assessing diffusion and release of the API. Both the first and second types can be used in thermosensitive ISSs for performing various tests, provided that the in vitro models are thermostatted.

The methods and equipment used in the pharmaceutical development of thermosensitive ISSs are listed in Table 2.

## 5. Conclusions

This review provides a comprehensive analysis of modern in situ thermosensitive systems for targeted drug delivery.

It is evident that thermosensitive polymers, especially those exhibiting a lower critical solution temperature (LCST) in the physiological range, represent a highly promising and versatile platform for the creation of “smart” drug delivery systems suitable for targeted delivery to various organs and tissues, both in physiological and pathological conditions. A detailed analysis of key polymer classes—including synthetic block copolymers (e.g., poloxamers, PLGA–PEG–PLGA) and modified natural polysaccharides (e.g., chitosan/β-glycerophosphate, xyloglucan)—highlights the availability of a diverse set of tools for composition developers, each with its own advantages and limitations.

It should be noted that the successful development of such systems must be based on a deep understanding of the underlying thermodynamic principles, primarily described by the Flory–Huggins theory, which explains the temperature-dependent sol–gel transition.

However, converting this theoretical knowledge into a viable pharmaceutical product is fraught with difficulties. The key problem is the sensitivity of critical quality parameters of delivery systems—such as the magnitude and stability of gel formation temperature, gel strength, and mucoadhesion—to both the polymer composition itself and external factors.

The nature and concentration of the active substance and excipients can significantly alter these parameters, highlighting the need for a science-based approach that evaluates the final formulation containing the active ingredient, rather than merely placebo compositions.

In addition, the existing range of in vitro, ex vivo, and in vivo assessment methods lacks uniform standardization, which leads to reproducibility issues and makes it difficult to directly compare the results of different studies. The development of biologically relevant, standardized, and validated methodologies, potentially including advanced in vitro models that mimic the multifactorial environment of the site of application, is important for improving the predictive power and success of preclinical studies.

In conclusion, thermosensitive ISSs have enormous potential to improve drug therapy through prolonged, targeted release and increased treatment adherence. Future efforts should focus on establishing reliable databases of well-characterized polymer matrices, optimizing compositions with a clear understanding of composition–property relationships, and implementing standardized, biologically relevant analytical methods. A consistent resolution of these issues will accelerate the pharmaceutical development of thermosensitive ISSs, paving the way for a new generation of effective and safe targeted drug delivery systems.

## Figures and Tables

**Table 1:** Comparative table of polymers for the development of in situ systems (ISSs).

Polymer Class/Specific Example	LCST/Gelation Temperature Range, °C	Mechanism of Gelation	Key Advantages	Main Limitations and Risks	Regulatory Status/Biocompatibility
Poloxamers (P407, Pluronic^®^ F127)	20–30 (concentration-dependent) [21,132]	Micellization and packing of micelles into an ordered/crystalline lattice [34,106]	High biocompatibility, ease of use, commercial availability, reversibility [21,133]	Low gel mechanical strength, rapid erosion, potential toxicity at high doses/concentrations, non-biodegradable [21,134]	FDA-approved as an excipient (Pluronic^®^ F68) and in medical devices. Extensive history of safe use [21]
PLGA–PEG–PLGA (triblock copolymer)	27–37 [37,91]	Micellization and subsequent temperature-induced packing of micelles due to dehydration of the PEG coronas [91,94]	Biodegradability, tunable degradation kinetics, prolonged release, good loading capacity [37,89]	Complex synthesis with risk of irreproducibility, autocatalytic acidification of the microenvironment upon degradation, influence of the drug on the LCST [135]	PLGA and PEG are approved individually. The specific triblock copolymer is a new excipient requiring a full safety dossier [136]
Chitosan/β-glycerophosphate (Chit/β-GP)	32–37 [60,137]	Physical crosslinking via reduced chitosan solubility: charge shielding, increased ionic strength, hydrogen bonding [60,137]	Biocompatibility, biodegradability, inherent mucoadhesiveness and antimicrobial activity of chitosan [58,59]	High variability in chitosan properties, sterilization challenges, gel is not reversible, risk of immune response [65,66]	Chitosan is approved as a wound biomaterial. The Chit/β-GP combination is a novel system requiring full registration [136]
PEGylated chitosan (PEG-g-chitosan)	30–38 [68]	Aggregation due to dehydration of PEG chains and hydrophobic segments of chitosan upon heating [64,68]	Solves the solubility issue of chitosan at neutral pH, combined properties (bioadhesion + stealth effect) [64,68]	Heterogeneity of the product during synthesis, potential reduction in mucoadhesion, risk of PEG immunogenicity [69,71]	A new chemical entity (NCE). Requires full toxicological and immunological profiling according to EMA/CHMP/SWP/372/01
Deglycosylated xyloglucan (Deg-XG)	28–34 [75]	Association of polymer chains through hydrogen bonding and hydrophobic interactions following removal of galactose side chains [73,75]	Natural origin, good mucoadhesion, biocompatibility, tunable LCST via degree of enzymatic treatment [73,76]	High variability of raw material, difficulty in controlling the enzymatic process, limited mechanical strength [73,74]	A novel biopolymeric excipient. Requires proof of non-immunogenicity and source standardization according to EMA/CHMP/SWP/372/01
Poly-N-vinylcaprolactam (PNVCL)	25–35 [114,116]	Coil-globule phase transition through dehydration of amide groups and enhanced hydrophobic interactions [114]	High biocompatibility, absence of toxic hydrolysis products (vs. PNIPAAm), tunable LCST via copolymerization [113,114]	Relatively high monomer cost, difficulty in obtaining polymer with narrow MWD, limited data on long-term in vivo degradation [114]	Active preclinical research. Considered a promising replacement for PNIPAAm. Full regulatory package is not available
Poly(2-oxazoline)s (POxs), e.g., PEtOx-PPropOx-PEtOx	25–65 (highly tunable) [123]	Depends on architecture. For amphiphilic triblocks—micellization and packing. For homopolymers—dehydration of side chains [123,138]	High biocompatibility, pronounced “stealth effect”, chemical versatility, ability to achieve narrow MWD [139]	Patent restrictions, more complex and expensive synthesis compared to poloxamers, limited industrial availability [140]	A new polymer class. PEtOx is the most studied. Requires accumulation of safety data for medical application
Elastin-like polypeptides (ELPs)	30–60 (precisely programmable) [125,141]	Reversible aggregation of polypeptide chains upon reaching the genetically programmed phase transition temperature [141,142]	Complete biodegradation to amino acids, low immunogenicity, precise design of properties (LCST, bioactivity) [21,142]	Very high production cost (heterologous expression), potential challenges with protein stability during storage [143,144]	Research stage. Promising for targeted therapy. The regulatory pathway will be complex due to its recombinant nature
Carboxylated agarose	~10–50 (dependent on degree of substitution) [79,145]	Weakening of hydrogen bonds in the agarose double helix and introduction of pH-dependent carboxyl groups, which shifts the gelation temperature [79]	Combined thermo- and pH-sensitivity, natural origin, possibility for ionic crosslinking [146]	Reproducibility of the degree of carboxylation, moderate mechanical strength, risk of plant-based impurities [147]	Natural agarose has GRAS (Generally Recognized as Safe) status as a food additive. The modified form is a new material for pharmacy
Aminated guar gum	30–40 [148]	Dehydration and association of chains enriched with amino groups, which also provide cationic mucoadhesion [148]	Combination of thermosensitivity and enhanced mucoadhesion, biocompatibility, antimicrobial potential [148]	Control of the degree of amination, potentially high solution viscosity, theoretical risk of immune response	Guar gum is approved as an excipient. The aminated form requires investigation as a new excipient
Poly(oligoethylene glycol)-based polymers (POEGMA and analogs)	20–90 (widely tunable) [100,111]	Dehydration of OEG side chains and aggregation of hydrophobic hydrocarbon backbones of macromolecules [100,111]	Fine-tuning of LCST by side chain length, good PEG-derived biocompatibility, possibility for photo/chemical crosslinking [100,102,111]	Complex and expensive synthesis with controlled architecture, risk of PEG immunogenicity with long-term use [71,110]	New synthetic polymers. Require a full registration dossier as new excipients (EMA/CHMP/QWP/396951/06 and EMA/CHMP/SWP/372/01)

**Table 2:** Methods and equipment used in the pharmaceutical development of temperature-responsive ISSs.

Subject	Characteristic/Parameter	Control Method	Equipment/Standard	Requirements/Quality Criteria
API	1.1 Solubility	General Pharmacopeia Monograph “Solubility” (State Pharmacopeia XV)	In accordance with pharmacopeial requirements	–
Polymer Matrix	2.1 Phase Transition Temperature (T_sol/gel_)	2.1.1 Heating in a Water Bath with Visual Control/Viscosity Measurement	Water bath, thermocouple, magnetic stirrer, viscometer, stopwatch	>32 °C (depends on the site of administration and pathology)
		2.1.2 Rotational Viscometry (USP NF <912>, EP 2.2.10)	Rotational viscometer (Brookfield, “cylinder in cylinder” type) with thermostat	–
		Differential Scanning Calorimetry (DSC)	Differential scanning calorimeter	–
	2.2 Gelation Time	2.2.1 “Inverted Test Tube” Method with Heating in a Water Bath	Water bath, thermocouple, magnetic stirrer, viscometer, stopwatch	<5 min (depends on the route of administration and in situ clearance)
		2.2.2 Rotational Viscometry with Time Control (USP NF <912>)	Rotational viscometer (Brookfield) with thermostat	–
	2.3 Viscosity (Before/After Sterilization)	Rotational Viscometry (USP NF <912>, EP 2.2.10)	Rotational viscometer (Brookfield, “cylinder in cylinder” type) with thermostat	SD < 3% (relative change after sterilization)
	2.4 Gel Stability (Long Term)	Statistical Analysis of T_sol/gel_ and Viscosity Reproducibility Over Time	Statistical Software (Any versions available to the researchers)	SD < 5% based on weekly measurements over ≥6 months
	2.5 Mucosal Adhesive Strength	2.5.1 Ex Vivo Mucosa Slip Test	Ex vivo mucosa, two-plate system	To be determined based on the testing protocol and intended site of application
		2.5.2 Mucin Detachment Test	Mucin solution	–
		2.5.3 Rotational Viscometry on a Mucin Model	Rotational viscometer (“cylinder in cylinder” or “plate–plate”) with a mucin model	–
		2.5.4 Measurement on a Texture Analyzer	Texture analyzer	–
	2.6 Biocompatibility	2.6.1 Cytotoxicity Assessment [163]	Cell culture, equipment for MTT assay	Cell viability > 70%
		2.6.2 Hemolysis Test [164]	Spectrophotometer	Hemolysis < 5%
		2.6.3 Intracutaneous Reactivity Assessment [165]	Standard equipment for in vivo tests	Absence of signs of irritation
ISS	3.1 pH	3.1 pH Potentiometry (SP RF XV, General Pharmacopoeia Monograph “Ionometry”)	pH meter	5.0–7.5 (depending on the site of application)
	3.2 Release Profile of API	3.2.1 Equilibrium Dialysis Method	Equilibrium dialysis apparatus	Compliance with the target release profile
		3.2.2 In vitro release test using Franz Cells	Franz cells, spectrophotometer or HPLC for analysis	–
	3.3 Quantitative Determination of API	Quantitative determination by an appropriate method (HPLC with a suitable detector) in accordance with USP NF <621> or EP 2.2.29	HPLC with UV, fluorescence, or mass spectrometric detector	--
	3.4 Sterility	3.4.1 Microbiological Control (SP RF XV, “Sterility”)	Laminar flow hood, incubator	Absence of microbial growth
		3.4.2 Test for Bacterial Endotoxins (LAL Test)	Spectrophotometer/fluorimeter	<0.25 EU/mL (for injectable dosage forms)
	3.5 Viscosity at Administration	Temperature Rotational Viscometry (USP NF <912>)	Rotational viscometer with thermostat	Depends on the route of administration (e.g., for injections 10–1000 mPa·s)
Comparative Evaluation	4.1 Comparison with Commercial Analogs	Comparative Analysis of T_sol/gel_, Gelation Time, Release Profile, Adhesive Strength	Equipment from Section 2 and Section 3	Quantitative comparison of key parameters to justify advantages
	4.2 Determination of Gelation Mechanism	4.2.1 IR Spectroscopy	IR spectrometer	Identification of key interactions (hydrogen bonding, hydrophobic interactions, phase transitions)
		4.2.2 NMR Spectroscopy	NMR spectrometer	–
		4.2.3 Oscillatory Rheometry	Rheometer	–

## Data Availability

The original data presented in the study are openly available in international online databases (Scopus, PubMed, Scholar Google, etc.).
